# Alterations in calmodulin-cardiac ryanodine receptor molecular recognition in congenital arrhythmias

**DOI:** 10.1007/s00018-022-04165-w

**Published:** 2022-02-08

**Authors:** Giuditta Dal Cortivo, Carlo Giorgio Barracchia, Valerio Marino, Mariapina D’Onofrio, Daniele Dell’Orco

**Affiliations:** 1 Department of Neurosciences Biomedicine and Movement Sciences, Section of Biological Chemistry, University of Verona, Strada le Grazie 8, 37134 Verona, Italy; 2 Department of Biotechnology, University of Verona, Strada le Grazie 15, 37134 Verona, Italy

**Keywords:** Calcium, Calmodulin, Molecular dynamics, Allostery, Arrhythmia

## Abstract

Calmodulin (CaM), a ubiquitous and highly conserved Ca^2+^-sensor protein involved in the regulation of over 300 molecular targets, has been recently associated with severe forms of lethal arrhythmia. Here, we investigated how arrhythmia-associated mutations in CaM localized at the C-terminal lobe alter the molecular recognition with Ryanodine receptor 2 (RyR2), specifically expressed in cardiomyocytes. We performed an extensive structural, thermodynamic, and kinetic characterization of the variants D95V/H in the EF3 Ca^2+^-binding motif and of the D129V and D131H/E variants in the EF4 motif, and probed their interaction with RyR2. Our results show that the specific structural changes observed for individual CaM variants do not extend to the complex with the RyR2 target. Indeed, some common alterations emerge at the protein–protein interaction level, suggesting the existence of general features shared by the arrhythmia-associated variants. All mutants showed a faster rate of dissociation from the target peptide than wild-type CaM. Integration of spectroscopic data with exhaustive molecular dynamics simulations suggests that, in the presence of Ca^2+^, functional recognition involves allosteric interactions initiated by the N-terminal lobe of CaM, which shows a lower affinity for Ca^2+^ compared to the C-terminal lobe in the isolated protein.

**Supplementary Information:**

The online version contains supplementary material available at 10.1007/s00018-022-04165-w.

## Introduction

**Fig. 1:**
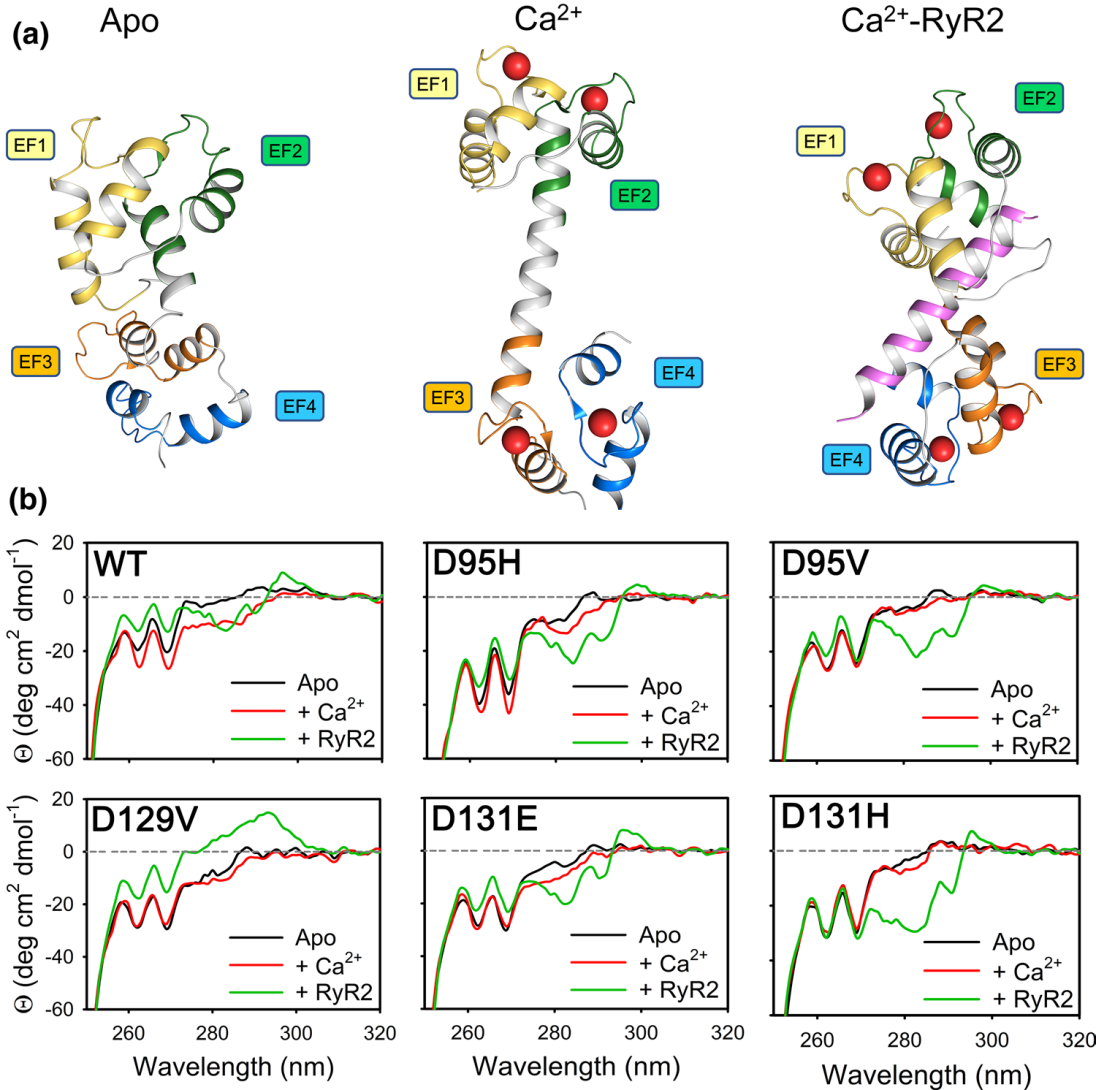
Structural analysis of CaM variants under different conditions. **a** Structural representation of CaM in the apo form (left, PDB: 1DMO [40]), Ca^2+^-bound (middle, PDB: 1CLL [41]) and complexed with RyR2 peptide (right, PDB: 6Y4O [15]). EF-hand motifs and RyR2 peptide (in pink) are represented as cartoon, Ca^2+^ ions are represented as red spheres. **b** Near UV CD spectra of 50 μM CaM variants in the presence of 0.5 mM EGTA (black lines) and after sequential additions of 1 mM Ca^2+^ (red lines) and 100 μM RyR2 (green lines)

Calmodulin (CaM) is a small (17 kDa) ubiquitously expressed Ca^2+^-sensor protein encoded by three genes (*CALM1-3*) located in different chromosomes of the human genome, all expressing the same amino acid sequence. The binding of up to four Ca^2+^ ions to the helix-loop-helix EF-hand motifs in CaM triggers a conformational change that exposes hydrophobic residues and allows optimal interactions with over 300 molecular targets [1]. The four functional EF-hands are organized in two distinct globular domains connected by a flexible linker region which, among possible conformations, can fold in α-helix, thus inducing CaM to acquire the typical dumbbell shape in the Ca^2+^-bound state (Fig. 1). In the absence of the target, the C-terminal domain (constituted by the EF3 and EF4 motifs) binds Ca^2+^ with higher affinity (*K*_*d*_ ~ 1 ﻿μM) compared to the N-terminal domain (EF1 and EF2; *K*_*d*_ ~ 10 μM). Positive cooperativity of Ca^2+^-binding occurs within each domain, but no inter-domain cooperativity has been detected in the absence of a target [2].

The extremely high structural plasticity of CaM [3] and the free-energy coupling between the processes of Ca^2+^- and target-binding induce significant changes in the cooperativity of Ca^2+^-binding in the CaM-target complex [4–6], which makes this system extremely sensitive in sensing even subtle changes in intracellular Ca^2+^ concentration. This mechanism enables a high selectivity and specificity of CaM mediated-target activation, as it allows fine regulation of a great variety of biochemical processes such as cytoskeleton remodeling, cellular mobility, proliferation, apoptosis, ion transport and protein folding [7, 8]. Among these processes, CaM participates in the Ca^2+^-induced Ca^2+^ release (CICR) mechanism in cardiomyocytes, a fundamental step for the regulation of the excitation–contraction process [9]. During cardiomyocyte depolarization, the opening of voltage-dependent Ca^2+^ channels leads to an increase in cytoplasmic Ca^2+^ concentration, thereby providing the signal for the opening of Ryanodine receptor isoform 2 (RyR2) [9, 10]. As a consequence, a massive Ca^2+^ release from the sarcoplasmic reticulum (SR) occurs, generating the actin- and myosin-mediated muscle contraction. RyR2 is per se﻿ capable of binding Ca^2+^, but it is the interaction with CaM that enables a precise regulation of the channel inhibition, which strictly depends on the concentration of free Ca^2+^ [9, 10].

The redundancy of genes encoding CaM in the human genome, its high conservation among the reigns and its promiscuity in terms of molecular partners suggest that missense mutations in the genes encoding CaM be deleterious and probably incompatible with life. However, in 2012 two missense mutations in CaM were found to be associated with Catecholaminergic Polymorphic Ventricular Tachycardia (CPVT) and long QT syndrome (LQTS) [11], both characterized by heart failure and sudden cardiac death. To date, 17 point mutations are known to be associated with cardiac diseases (see ref [12] for a recent review), which contributed to define a new branch of pathology termed calmodulinopathy, whose clinical phenotype affects mostly young individuals [13].

**Fig. 2:**
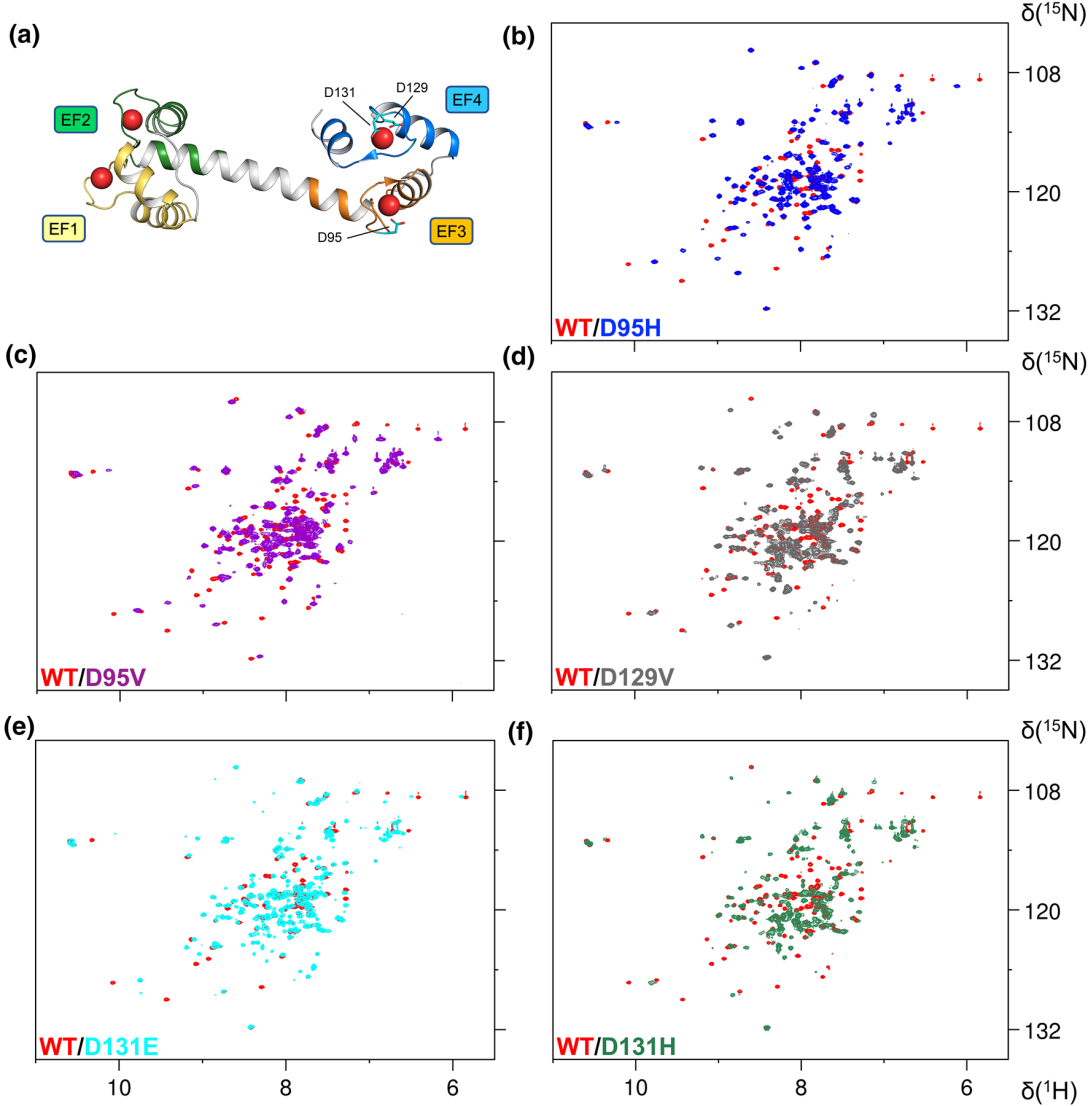
Bidimensional NMR spectra of CaM variants. **a** Three-dimensional structure of Ca^2+^-CaM (PDB entry: 1CLL [41]). Mutated residues D95, D129 and D131 are shown as cyan sticks on the protein structure. **b**–**f** Overlay of the ^1^H-^15^ N HSQC NMR spectra of 100 µM ^15^ N-WT CaM (red) and its variants in the presence of 2 mM Ca^2+^ ions

The ubiquitous expression of CaM in all cell types on one hand, and the specific expression of RyR2 in cardiomyocytes on the other, suggest that the molecular mechanism underlying the onset of the disease is related to perturbed recognition between CaM and RyR2. This interaction has been extensively characterized by X-ray crystallography, Nuclear Magnetic Resonance (NMR), fluorescence anisotropy (FA) [14–18], and, more recently, cryogenic electron microscopy (CryoEM), which revealed key structural features of RyR2-CaM interactions [19]. Despite the many aspects investigated by these contributions, some key features of the recognition process remain to be clarified. For example, information is missing as to the kinetics of RyR2-CaM recognition, which is expected to play an essential role in the dynamics of Ca^2+^-signaling in cardiomyocytes, as it could lead to mutation-dependent alterations of the CICR process. Moreover, it has been established for wild type CaM that the lack of interaction between the globular domains reflects in the absence of inter-domain cooperativity of Ca^2+^-binding [2]; however, it is unclear whether the four EF-hands may show long-range allosteric communication with one another and with the target in the presence of RyR2, which could result in a differential regulation of the channel by WT and mutant CaM. In this work, we clarify some of these aspects by providing a structural, kinetic and thermodynamic comparison of the recognition process between the CaM-interacting region of RyR2 and both wild type CaM and five missense variants associated with LQTS localized in the C-terminal lobe (Fig. 2a), specifically in EF3 (D95H, D95V) [20] and EF4 (D129V [21], D131E [22], and D131H [23]; note that the nomenclature of CaM variants in this work is based on the mature protein that lacks the Met in position 1).

## Materials and methods

### Plasmids and peptide

The optimized plasmids for the expression and purification of WT and mutated CaM forms have been purchased from Genscript. The cDNA of human calmodulin (Uniprot entry: P0DP23) was cloned into a pET24( +) vector (resistance to kanamycin) and point mutations were checked by sequencing. A 6xHis tag was added to the N-terminal, followed by a Tobacco Etch Virus (TEV) protease recognition/cleavage site (thus resulting in the construct MKHHHHHHPMSDYDIPTTENLYFQGA-CaM). The RyR2 peptide, encompassing the region R3581-L3611 of the RyR2 channel, had the following sequence: RSKKAVWHKLLSKQRKRAVVACFRMAPLYNL and was purchased from Genscript (purity > 95%, checked by mass spectrometry and HPLC). The lyophilized peptide was initially resuspended in pure bi-distilled water at a concentration of 400–500 µM (based on its mass determined by amino acid analysis performed by Genscript) and stored at − 80 °C until use. Before each experiment, the peptide was diluted in a working buffer and quantified according to its absorbance at 280 nm (*ε* = 5690 M^−1^ cm^−1^).

### CaM expression and purification

Transformed BL21 DE3 cells were grown at 37 °C until the OD_600_ reached 0.6, at which point the expression was induced by the addition of 1 mM Isopropyl-*β*-D-galactopyranoside (IPTG). Cells were harvested after 4 h by centrifugation at 4 °C, 5500 × g for 20 min and the obtained pellet was resuspended using lysis buffer (50 mM TRIS pH 8, 0.5 M NaCl, 10 mM imidazole) containing lysozyme (0.1 mg/mL), DNAse (5 U/mL) and proteases inhibitor cocktail (Roche). After 30 min incubation at 30 °C and 12 × 10 s sonication cycles on ice, the soluble and insoluble fractions were separated by centrifugation at 16,000 × g at 4 °C, 30 min. The soluble fraction was directly loaded on a His-trap FF Crude column (GE) previously equilibrated, and the His-tagged CaM was obtained by one-step elution using 500 mM imidazole. The eluted His-tagged protein was dialyzed against 20 mM TRIS, 150 mM NaCl; an aliquot was washed against 20 mM HEPES, 150 mM KCl and flash-frozen to be used for surface plasmon resonance measurements. The rest of the His-tagged protein was incubated with TEV-protease (Promega, 1U per 400 µg) overnight at 25 °C in the presence of 1 mM fresh DTT. Tag-free CaM was purified by reverse immobilized metal affinity chromatography (IMAC) collecting the column flow-through. The purity of the obtained protein was tested by sodium dodecyl sulphate polyacrylamide gel electrophoresis (SDS-PAGE). Protein was quantified by Bradford assay using a calibration line optimized for human CaM based on amino acid analysis (Alphalyze), flash-frozen in liquid nitrogen and stored at − 80 °C until use.

^15^N-labeled CaM samples for NMR experiments were obtained using M9 minimal medium supplemented with 1 g/L ^15^NH_4_Cl (CIL) as the sole source of nitrogen, using the same protocol as for the untagged protein.

### Native polyacrylamide gel electrophoresis (native PAGE)

The capability of CaM variants to interact with RyR2 peptide was assayed by PAGE under native, non-denaturing conditions. CaM and RyR2 were combined at different stoichiometric ratios (1:0 to 1:2 CaM:RyR2) using 20 mM TRIS, 150 mM KCl, 1 mM DTT, 340 µM Ca^2+^ as working buffer. After 10 min incubation at 25 °C, the samples were loaded on 15% polyacrylamide continuous gels (390 mM TRIS pH 8.8), run at 200 V in 25 mM TRIS pH 8.8, 192 mM glycine buffer for 45 min and Coomassie Blue-stained.

### Limited proteolysis

Limited proteolysis experiments were performed incubating 0.33 μM trypsin (Sigma) with 20 μM CaM variants (ratio 1:60) at 25 °C in the presence of 2 mM Ca^2+^ or EGTA, using 20 mM TRIS, 150 mM KCl, 1 mM DTT as working buffer. First, a time scan was performed for 2 h (see Fig. S1a and S1b) using WT CaM, to monitor the incubation time that provided the best resolution of proteolytic patterns under both conditions, thus allowing the clearest comparison of the cleavage fragments. Then, the experiment was repeated for all the CaM variants; samples were collected after 10 min digestion and loaded on a 15% polyacrylamide gel, run for 45 min at 200 V and Coomassie Blue-stained.

### Circular dichroism

Circular dichroism measurements were performed to assess secondary and tertiary structure properties. Experiments were performed using a Jasco J-710 spectropolarimeter supplied with a Peltier-type cell holder. Five accumulations were recorded for each condition, and the results were the average spectra. Proteins and peptide were quantified before each measurement by Bradford assay and A_280_, respectively. Far UV measurements were collected between 200 and 250 nm using 10 µM CaM variant after sequential additions of 300 µM EGTA and 600 µM Ca^2+^, in the absence and in the presence of 20 µM RyR2. Near UV measurements were collected in the 250–320 nm range using 50 µM CaM after sequential additions of 0.5 mM EGTA, 1 mM Ca^2+^ and 100 µM RyR2. Quartz cuvettes with 0.1 and 1 cm length path were used for far and near UV spectra, respectively. The spectrum of 20 mM TRIS, 150 mM KCl, 1 mM DTT was collected and considered as blank. Spectra were collected at 25 °C, setting time response at 4 s, data pitch 1 nm, bandwidth 5 nm.

### Dynamic light scattering (DLS)

The hydrodynamic radius of WT and CaM variants was assayed by dynamic light scattering (DLS) using a Zetasizer Nano-S (Malvern Instruments) and disposable polystyrene low volume cuvettes. The parameters for size estimation were set as follows: refraction index (RI) of the sample 1.450, 1.330 for the dispersant (water); viscosity was set to 0.8872 cP. Thirty micromolar CaM was used in each DLS experiment, which ran for approximately 2 h at 25 °C, in the presence of 1 mM EGTA and after the addition of 2 mM Ca^2+^. Protein solutions as well as EGTA and Ca^2+^ stocks were passed through a 20 nm filter (Anotop, Whatman) before each experiment. Data were analyzed using the Multiple Narrow mode (high resolution). Figure S3 reports DLS profiles together with the mean ± standard error of the mean (SEM) of each main peak.

### Nuclear magnetic resonance (NMR)

NMR spectra were recorded at 600 MHz on a Bruker Avance III spectrometer, equipped with a TCI CryoProbe or on Bruker Avance NEO spectrometer equipped with a Cryo probe Prodigy TCI. The spectra were recorded at 25 °C. Typical ^1^H-^15^N heteronuclear single quantum coherence (HSQC) spectra were performed with a data matrix consisting of 2048 (F2, ^1^H) × 256 (F1, ^15^N) complex points, spectral windows of 8417.509 Hz (^1^H) × 2432.717 Hz (^15^N), 16 transients, and 1.2 s relaxation delay. Unless otherwise specified, 2D experiments were run on samples at a protein concentration of 100 µM with 2 mM Ca^2+^ and with 2 mM Ca^2+^ and 200 µM RyR2 peptide, dissolved in 20 mM TRIS pH 7.5, 150 mM KCl, 7% D_2_O buffer.

NMR titration experiments were run on samples with a protein concentration of 300 µM in 20 mM TRIS, pH 7.5, 150 mM KCl, 0.4 mM EGTA, 7% D_2_O buffer with Ca^2+^ ions added stepwise from a concentrated stock solution (100 mM). The following protein/ligand rations were analyzed by SOFAST-HMQC (Band-Selective Optimized Flip Angle Short Transient) spectra: 1:0, 1:0.5, 1:1.5, 1:7, 1:20. Each experiment was performed with a data matrix of 960 (F2, ^1^H) × 160 (F1, ^15^N) complex points, spectral windows of 8196.722 Hz (^1^H) × 1824.533 Hz (^15^N), 8 transients, and 0.1 s relaxation delay. All data were processed and analyzed using TOPSPIN 4.1.1 (Bruker, Karlsruhe, Germany).

### Surface plasmon resonance (SPR)

SPR measurements were performed at 25 °C using a SensiQ Pioneer instrument and His-Cap Biosensor chips (Fortèbio). SPR chips were prepared for protein immobilization according to manufacturer’s instructions and 1800–2000 Resonance Units (RU; 1 RU = 1 pg mm^−2^) of each variant were immobilized using 20 mM HEPES/KOH pH 7.5, 150 mM KCl, 0.005% Tween 20 as running buffer. After an overnight wash cycle (flow rate: 5 μL/min) to wash off the unbound protein, 100 μM DTT and 5 mM CaCl_2_ were added to the running buffer. Titration experiments were designed to inject increasing amounts of RyR2 (diluted in the same running buffer), ranging from 250 nM to 3 µM for 60 s and following the dissociation for 300 s. Flow rate was set at 20 µL/min and each injection was performed in triplicate. Data were fitted to a 1:1 Langmuir model. First, dissociation curves were fitted to a single exponential function to obtain *k*^off^ values. The *k*^off^ values were then used to calculate the association rate constant *k*^on^ according to a pseudo-first-order kinetic scheme. Each kinetic parameter was the average of 8 to 15 values from independent injections. Data reported in Fig. 4 represent the mean and the SEM for each parameter.

### Isothermal titration calorimetry (ITC)

ITC measurements were performed using a MicroCal PEAQ instrument at 25 °C. Seven-to-ten micromolar CaM was titrated with RyR2 (125 µM peptide loaded in the syringe). Thirty injections of 1 µL were performed, setting the stirring speed to 750 rpm, with 150 s interval between subsequent injections. Data refer to 3–6 replicas for each injection. Titrations from all CaM variants could be fitted by the one set of sites binding model except for D129V, whose titration did not lead to saturation in the same range of relative CaM:peptide concentrations. Titrations performed at lower CaM:peptide ratios reached saturation (Fig. S13d); however, the observed isotherm was biphasic and could not be fitted by the same fitting model used for other CaM variants.

Number of binding sites (*N*), dissociation constant (*K*_*D*_) and enthalpy changes (Δ*H*) were the experimental parameters (Table S2) obtained by the best performing fitting model (one set of sites) and they were used for the calculation of Δ*G*, entropy changes (Δ*S*) and ΔΔ*G* according to the following formulas:

$$\Delta G=RT ln {K}_{D}=\Delta H-T\Delta S$$

$$\Delta \Delta G= \Delta {G}_{mut}-\Delta {G}_{WT}$$

## Molecular modeling of CaM-RyR2 complex

The molecular modeling of Ca^2+^-loaded human CaM-RyR2 complex was obtained using as starting structure the high-resolution complex corresponding to the Protein Data Bank entry 6Y4O [15] (resolution 1.84 Å), which contains the crystallographic structure of CaM bound to the RyR2 peptide encompassing the region from S3582 to M3605. Structures were prepared using the BioLuminate module (v. 4.0.139) from the chemical simulation suite Maestro (v. 12.5.139, Schroedinger) following the *Protein preparation* tool pipeline. Briefly, bond orders (including zero-order bonds to Ca^2+^-ions) were assigned based on the Chemical Components Dictionary database, original H atoms were replaced with BioLuminate-generated hydrogens, missing side chains and loops (residues 77–80) were modeled using Prime. The model was then refined by sampling water orientation, protonation of ionizable residues at pH 7.5 using PROPKA, H-bond optimization and removal of water molecules. In silico﻿ variants, D95H, D95V, D129V, D131E and D131H of human CaM were introduced using the BioLuminate *Mutate residue* tool by selecting the highest scored non-clashing sidechain rotamer.

### Molecular Dynamics simulations and reproducibility analyses

All-atom Molecular Dynamics (MD) simulations of human CaM-RyR2 peptide complexes were run using GROMACS v. 2020.3 simulation suite [24] and CHARMM36m forcefield [25]. Simulations were performed in a dodecahedral box (system size 35,849–35,858 atoms) after neutralization of the net charge with 150 mM KCl, energy minimization and sequential 2 ns equilibration in NVT and NPT ensembles as previously detailed [26]. Productive MD simulations consisted of 4 × 300 ns replicas at constant temperature (310 K) and pressure (1 atm), the conformational space sampling was assessed by means of Root-Mean Square Inner Product (RMSIP) of the Essential Subspace (ES) and Linear Discriminant Analysis (LDA) of the projections of the trajectories onto the first two principal components (PC) as described in Refs.[27, 28]. Briefly, the covariance matrix of the position of Cα atoms allowed the calculation of the eigenvalues and eigenvectors representing the largest collective motion of the systems. The 300 ns trajectories were concatenated, projected onto the 2 PC with the highest eigenvalues and subjected to LDA, a classification method that identifies a lower-dimensional space that minimizes intraclass distances and maximizes interclass distances. Additionally, the ES represented by the first 20 PC of each isolated and concatenated trajectory was compared using the *RMSIP* calculated as follows:

$$RMSIP = \left( {\frac{1}{S}\sum\limits_{n = 1,\;m = 1}^{S} {\left( {v_{n}^{i} \cdot v_{m}^{i} } \right)^{2} } } \right)^{{{1 \mathord{\left/ {\vphantom {1 2}} \right. \kern-\nulldelimiterspace} 2}}}$$

where *v*_*n*_^*i*^ and *v*_*m*_^*j*^ represent the *n*th and *m*th eigenvectors of the ES of replicas *i* and *j*, respectively, consisting of *S* PC (20 in this case).

The consistency of four replicas of the 300 ns MD trajectories was assessed by comparing the directions of collective motions of each simulation with the concatenated 1.2 µs trajectory by means of principal component analysis (PCA) of C*α* and LDA applied to the projection of the trajectories onto the first 2 PC (Fig. S14), which highlighted that most of the conformations could be assigned to different replicas, as shown by the substantial overlapping of the density functions (Fig. S15). Similar results were obtained by comparing the RMSIP calculated on the first 20 PCs of each replica and the concatenated trajectories (Fig. S16), with average values higher than 0.8, thus implying the consistency of the conformational space sampled by the simulations. MD simulations were, therefore, reproducible and consistent and could thus be used for topological analyses.

The persistence of H-bonds, hydrophobic and electrostatic interactions within the protein-peptide complexes was monitored to filter out transient interactions and identify only those responsible for defining and maintaining their three-dimensional structure.

### Protein structure network analysis

The dynamic structural information provided by MD simulations was encoded in a Protein Structure Network (PSN) for each CaM-RyR2 peptide complex using PyInteraph [29], employing the same cut-off distance values and criterion for persistence threshold calculation as in Ref. [30]. Briefly, the percentage of frames in which the distance and angle constraints for non-covalent interactions (H-bonds, hydrophobic and electrostatic interactions) between couples of amino acids were satisfied was calculated by PyInteraph. If such persistence exceeded the threshold (*pT*, based on the size of the biggest hydrophobic cluster criterion [30]), the interaction was included in the PSN as an edge between the two nodes (representing the two amino acids). The degree centrality, defined as the number of edges incident upon a node, was calculated for each residue in the network to highlight which amino acids were entitled to the highest number of interactions and thus responsible for maintaining the correct folding and mediating intra/intermolecular communication. Communication between two amino acids of interest, regardless of how far apart they are within the protein’s tertiary structure, accounts for all the persistent hydrophobic, electrostatic, and H-bonds interactions required to transfer structural information such as binding status or conformational changes. The Communication Robustness index defined in Ref. [30] was employed to monitor differences in intramolecular communication among EF-hands (represented by E31, E67, E104 and E140) and between each EF-hand and the interface CaM residues showing persistent interactions with RyR2 peptide in the WT complex (E11, E15, A15, F19, E47, M72, K75, E84, A88, L112, E114, E120, E123, M124, E127, A128, M144 and M145).

CR is calculated as follows:

$$CR\left(AB\right)=\frac{nAB*pT}{l}$$

in which *pT* is the persistence threshold, *nAB* is the number of shortest paths connecting *A* and *B* residues and *l* is the length of such shortest paths.

Variations in hub degree shown in Fig. S15 refer to nodes with more than 6 interactions in the PSN of at least one of the 6 variants.

## Results and discussion

X-ray crystallography revealed a detailed picture of the interaction between WT CaM and RyR2 and showed that the structure of the LQTS-associated N53I variant, located at the N-terminal domain, only slightly differs from that of the WT [15]. Nonetheless, perturbation of the intramolecular dynamics was detected for the mutant, which reflected in an altered interaction with the RyR2 target [15]. Here, we investigated whether and how the arrhythmia-associated mutations localized in EF3 and EF4 may alter such interaction. We characterized the structural features of the D95V/H substitutions in EF3 and of the D129V and D131H/E substitutions in EF4 (Fig. 2a) at increasing levels of resolution, moreover, we probed whether dynamic and/or allosteric alterations occur in the interaction with the RyR2 peptide.

### Mutation-specific structural alterations of arrhythmia-associated CaM variants

To investigate whether mutations in CaM alter their exposure to proteases, CaM variants were digested using trypsin (see Experimental Section) in the absence and in the presence of Ca^2+^ (Fig. S1). The time dependence of the proteolytic digestion of WT CaM in the presence of EGTA (Fig. S1a) or Ca^2+^ (Fig. S1b) highlighted a clear stabilizing effect of the cation. When proteolysis was followed for 10 min for CaM WT and variants, different digestion patterns were detected. A comparison of the patterns for the EF3 and EF4 mutants in Ca^2+^-free conditions (Fig. S1c and S1e) highlighted the presence of either two (D95H/V) or three fragments (D131H/E, D129V). Interestingly, the charge-conservative mutation D131E resulted in a less stable protein, as judged by the almost complete disappearance of the undigested band. The addition of Ca^2+^ stabilized all the variants, though to a different extent. Digestion of EF4 variants (Fig. S1f) indeed generated two slightly bigger fragments (MW ~ 10–17 kDa) while EF3 variants showed three main proteolytic fragments at lower MW (8–13 kDa, Fig. S1d). Proteolysis, therefore, suggests that structural alterations depend on the site (EF-hand) of the mutation, and that Ca^2+^ exerts a stabilizing effect on all variants, although significantly smaller than for WT CaM.

The structural effects of amino acid substitutions in EF3 and EF4 were investigated by circular dichroism (CD) spectroscopy in the far and near UV spectral regions, to monitor potential alterations in protein secondary and tertiary structures, respectively. Figure 1b shows near UV CD spectra of CaM variants in the presence of saturating EGTA (black lines) and Ca^2+^ (red lines). Although CaM lacks tryptophan residues, absorbance changes in the 250–320 nm region due to other aromatic residues present in its structure (8 Phe and 2 Tyr) enable Ca^2+^-induced conformational changes to be monitored. Interestingly, all CaM variants showed slightly altered response to Ca^2+^-binding compared to the WT, both in terms of signal intensity and spectral shape. Indeed, WT CaM showed a more negative spectrum in the Phe and Tyr bands upon saturation with Ca^2+^, while almost overlapping spectra were recorded for D95V, D131H and D129V. Slight variations were observed in the Tyr band of D131E and D95H, which was in general more dichroic than the other variants.

Far UV CD spectroscopy highlighted EF-hand-specific effects of the pathogenic mutations on the secondary structure of CaM. In many calcium sensor proteins, Ca^2+^-binding is accompanied by an increase of the dichroic signal, which can be attributed to an increased α-helix content or to the achievement of a more compact structure [3, 26]. This was the case for WT CaM (Fig. S2a), whose far UV CD spectrum was typical of a mainly α-class protein, with the two minima at 208 and 222 nm shifting to more negative values upon addition of Ca^2+^. A similar pattern was observed also for the EF3-mutants (D95H and D95V, Fig. S2b and S2c), at odds with the EF4 variants D129V and D131H/E, which displayed a decrease in ellipticity upon Ca^2+^ addition (Fig. S2d, S2e and S2f) and a negative relative ellipticity at 222 nm (Δ*θ*/*θ*) (Table S1). A 14% increase of relative ellipticity was detected for WT CaM, accompanied by an increase of the *θ*_222_/*θ*_208_ ratio from 0.93 to 0.97, indicative of variations in protein secondary structure. A similar trend was observed for D95H and D95V CaM. Interestingly, when the mutation affected EF4 residues, Ca^2+^-binding led to negative Δ*θ*/*θ* changes. This, together with the generally smaller *θ*_222_/*θ*_208_ values (Table S1) suggests a loss of *α*-helix content and/or a less compact structure. Dynamic light scattering measurements (Fig. S3) showed that all CaM mutants display increased hydrodynamic radius compared to WT CaM, with the biggest variation (exceeding 3 nm) observed for the D95H/V EF3 variants. Variations in the hydrodynamic radius of Ca^2+^-sensors have been shown to be sensitive to even single point mutations, when these affect the exposure of hydrophobic patches [31], thus suggesting that the observed changes in far UV CD spectra may be partly due to alterations of protein compactness.

CaM mutants were also analyzed by nuclear magnetic resonance (NMR) spectroscopy to determine structural changes at the atomic level. The overlay of 2D heteronuclear ^1^H-^15^N HSQC spectra collected for WT CaM vs the different LQTS mutants in the Ca^2+^-free forms (Fig. S4) shows that the spectra are almost completely superimposable, with the exception of few peaks belonging to residues mutated or surrounding the mutations. This evidence suggests that the overall structure of CaM is only minimally impacted by the mutations in the Ca^2+^-free forms. Further analysis was then performed on WT CaM vs the different mutants in the Ca^2+^-bound forms. The ^1^H-^15^N HSQC spectra of all the mutants exhibited a degree of resonance dispersion and peak resolution equivalent to that of the WT (Fig. 2, S5 and S6), although the intensity was not homogeneous for all the peaks in the spectra. In addition, a fraction of the resonances in the spectra of each of the mutants were superimposable with equivalent resonances in the spectrum of WT CaM. The different position of many peaks in the ^1^H-^15^N HSQC spectra confirmed that all the mutants undergo substantial changes in the Ca^2+^-bound form with respect to the WT protein, in line with results from CD spectroscopy. The difference appeared larger for D131H, D95H and D129V, and it could be attributed to local conformational changes in the surrounding of the mutated residue, but it also extends to other less proximal regions (Figs. S5 and S6).

A close inspection of the downfield region in the HSQC spectra of the Ca^2+^-bound mutants, indicative of the conformation of the Ca^2+^-binding sites, showed that all mutations except D129V significantly perturbed the EF4 motif, with the peak of G134 showing a significantly lower intensity or chemical shift variation compared to the signal in the WT protein (Fig. S7). Although the overlap of the signals in some areas of the spectra prevents a clear interpretation, the peaks’ position suggests that mutations did not significantly perturb the Ca^2+^-bound form of EF1 and EF2 motifs (Figs. S5 and S6). A peculiar behavior was observed for D95V (Fig. S7c), where all the downfield peaks were slightly perturbed with respect to WT CaM, which could be attributed to the hydrophobic nature of the substitution rather than to its position. These observations are in line with previous studies showing the large conformational changes occurring on D95V CaM with respect to WT [32].

The position of the downfield peaks is only slightly perturbed in all analyzed variants (Fig. S7). However, the comparison of WT HSQC spectrum with that of EF4 mutants in Ca^2+^-bound forms revealed the presence of new, well-dispersed peaks (at 110–109 and 9–8 ppm in the ^15^N and ^1^H dimensions, respectively) that are indicative of a perturbation of the structure of these variants with respect to WT, in line with the two ensembles of CaM variants observed in far UV CD spectroscopy.

### Structural alterations of arrhythmia-associated CaM variants are Ca^2+^-dependent

To clarify the dynamics of structural changes induced by the mutations in EF3 and EF4, we performed Ca^2+^-titration NMR experiments. As already mentioned, the analysis of the chemical shift/intensity perturbation of the signals in the downfield region was not significant due to peak overlapping. We, therefore, analyzed the behavior of other residues, as reporters of the binding of Ca^2+^ to CaM mutants.

The chemical shifts of the peaks belonging to G33 in all CaM mutants were very similar to WT both in apo and Ca^2+^-bound forms (Fig. S8), suggesting that this area of the protein is minimally impacted by the mutations. However, the analysis of the titration experiments revealed that WT, D131H, and D95V variants displayed an intermediate to fast or fast exchange regime for G33, while D129V, D131E, and D95H displayed a slow-exchange regime (Fig. S9). This observation could suggest the presence of a long-range communication pathway between the two lobes of CaM, slightly perturbed by the mutations during the uptake of Ca^2+^ ions. As a further reporter of the Ca^2+^-CaM interaction, we analyzed G23 (in EF1) and G96 (in EF3) (Fig. S10). The former was unperturbed in all mutants, in the transition to Ca^2+^-CaM, whereas the latter was visible only in D131E in the Ca^2+^-bound form, in the same position as in the WT. This suggests that the mutations do not significantly alter the Ca^2+^-binding properties in EF1, while binding to EF3 appears to be equally perturbed regardless of the location of the mutation (EF3 or EF4).

It is also interesting to notice the peculiar behavior of residue G113, located between EF3 and EF4 (Fig. S11). In all CaM variants, G113 showed a slow exchange regime between the unbound and bound forms upon the addition of Ca^2+^. Conversely, in the apo form the position of the peak was the same in all CaM variants, while the final position of the peaks, upon addition of Ca^2+^, was found to depend on the mutated residue.

Notably, in mutants D95H and D95V the peak of G113 of the Ca^2+^-bound forms was observable only at a high metal:protein ratio. This analysis identifies a cluster for EF3 mutants, in line with results from far UV CD (Fig. S2 and Table S1).

### Structural insights into CaM-RyR2 interactions

The R3581-L3611 region of RyR2 is known to constitute a major interacting interface for the recognition of CaM [14, 33], therefore, we used a peptide encompassing this amino acid stretch to monitor CaM-RyR2 interaction for WT and mutant CaM. The formation of a CaM-RyR2 complex at 1:1 was observed using polyacrylamide gel electrophoresis, for all CaM variants (Fig. S12). At odds, the EF4 mutants showed the uncomplexed protein band even at double the RyR2:CaM molar ratio, suggesting a decreased affinity.

**Fig. 3:**
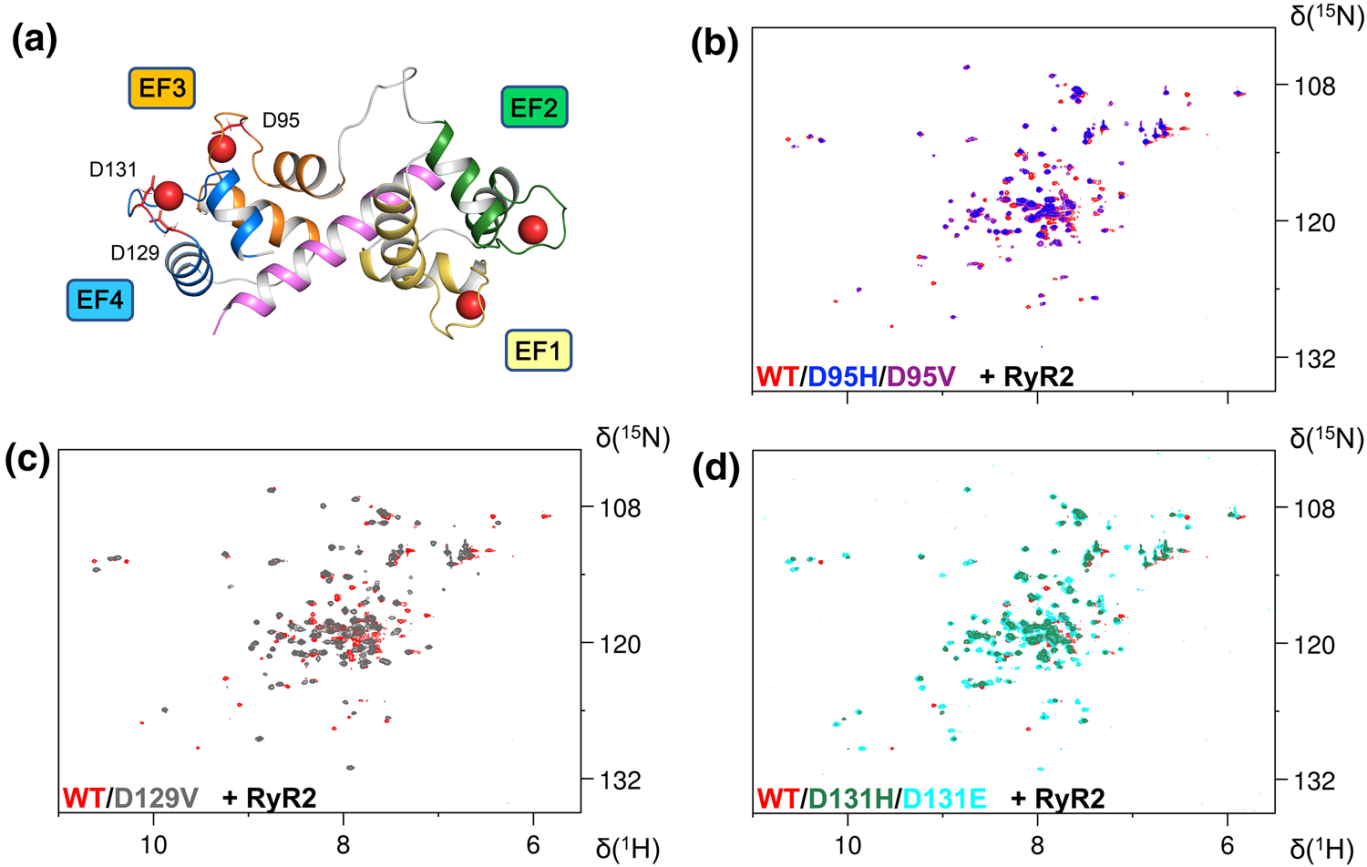
Structural effects of CaM-RyR2 interaction monitored by NMR spectroscopy. **a** Three-dimensional structure of Ca^2+^-loaded CaM in complex with RyR2 peptide (PDB: 6Y4O [15]). Mutated residues D95, D129 and D131 are shown as sticks. **b**–**d** Overlay of the 2D ^1^H-^15^ N HSQC NMR spectra of ^15^ N-WT CaM and its variants in presence of 2 mM Ca^2+^ ions and 2 eq. of RyR2 peptide

The binding of RyR2 to Ca^2+^-bound CaM variants was also investigated by NMR (Fig. 3). In all cases, only few peaks maintained their position in the ^1^H-^15^N HSQC spectra recorded after the addition of 2 equivalents of RyR2, thus showing that all mutants were able to bind the peptide. The significant chemical shift perturbations occurring for many peaks suggest that the RyR2 peptide imparts major structural changes to the Ca^2+^-bound CaM variants. This observation is in line with previously reported data on D95V CaM showing an altered conformation in the complex with a peptide of the CaV1.2 IQ domain [32]. Despite the significant differences in the spectra of WT, D95H and D95V CaM in their Ca^2+^-bound forms (Fig. 2), upon addition of RyR2 the three proteins displayed many peaks in the HSQC spectra with similar chemical shifts (Fig. 3). Similar conclusions could be drawn by analyzing the spectra of WT vs D131H and D131E in the presence of RyR2, thus suggesting a similar structure of the complexes that is fully in line with far UV CD spectroscopy. These data show that the addition of the RyR2 peptide to Ca^2+^-saturated CaM may dampen the structural differences observed in the spectra of EF3 and EF4 mutants (Fig. S2 and Table S2).

It is interesting to note that D129V CaM displayed a significant chemical shift perturbation upon addition of RyR2 (Fig. 3c), with a final conformation of the protein in the complex substantially different from that of the WT and other mutants, in line with results from near UV CD spectroscopy (Fig. 1b).

Taken together, these lines of evidence suggest subtle structural alterations of the CaM-RyR2 complex under saturating Ca^2+^, with a more prominent structural change observed for the D129V CaM variant.

### Thermodynamics and kinetics of the interaction between RyR2 and LQTS-associated CaM variants

Isothermal titration calorimetry (ITC) has been previously used to characterize the thermodynamics of WT and N53I [15] and F141L [16] arrhythmogenic variants CaM binding to a shorter version of the same RyR2 peptide used here (lacking the -LYNL sequence at the C-terminal) [34]. We performed ITC titrations of the RyR2 peptide on both WT and LQTS CaM mutants under saturating Ca^2+^ concentration. In line with previous studies, the binding isotherms were compatible with an exothermic process characterized by a 1:1 protein-peptide stoichiometry, and with mutation-specific affinity, as suggested by changes observed in the steepness of the transition (Fig. S13). For each CaM variant, the process was enthalpy-driven (Table S2) with unfavorable entropic contributions. All measured *K*_*D*_ values were in the low nM range (*K*_*D*_ = 8.6 nM for WT CaM, Table S2), substantially in line with previous determinations (*K*_*D*_ = 12 nM [1, 15] and 47 nM [34] for WT CaM; the slight difference can be attributed to the shorter peptide used in previous studies). Although all variants resulted in more negative enthalpic contributions compared to the WT, the effect on the stabilization of the complex (∆∆G) was mutation-dependent, ranging from 1.8-fold increase in affinity for D95V (corresponding to 0.35 kcal/mol stabilization) to 3.9-fold decreased affinity, corresponding to a 0.81 kcal/mol destabilization for D131H (Table S2). A peculiar behavior was observed for D129V, whose titration resulted in a biphasic trend that, unlike the other variants, could not be fitted to a simple thermodynamic model. This suggests a more prominent structural rearrangement of the protein-peptide complex, in line with NMR data (Fig. 3c) and near UV CD spectroscopy (Fig. 1b).

**Fig. 4:**
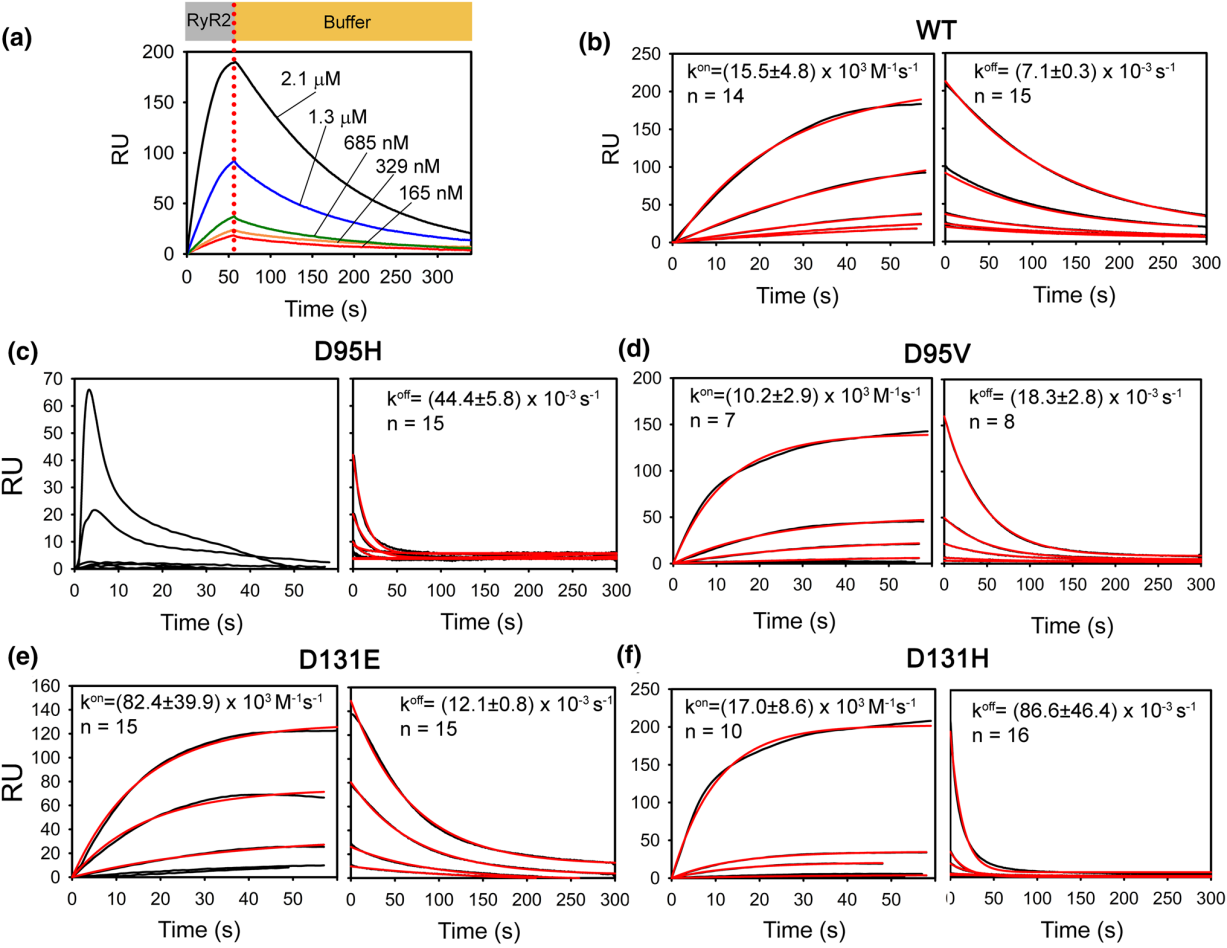
Surface Plasmon Resonance kinetic analysis of CaM-RyR2 interaction. **a** Example of titration with increasing concentrations of RyR2 (165 nM to 2.1 μM) injected on WT His-CaM immobilized on a His-Cap sensor chip. Association (grey box) and dissociation (orange box) phases were followed for 60 s and 300 s, respectively. Association and dissociation rate constants (*k*^on^ and *k*^off^) are reported in each panel. Experimental curves (black solid lines) are shown together with theoretical curves (red solid lines) after fitting to a 1:1 Langmuir binding model

To obtain kinetic information on CaM-RyR2 interaction at saturating Ca^2+^ levels under non-equilibrium conditions, we used Surface Plasmon Resonance (SPR) spectroscopy. Figure 4 displays exemplary sensorgrams obtained following injections of increasing amounts (165 nM to 2.1 μM) of RyR2 peptide on a sensor chip where similar levels of each CaM variant were homogenously immobilized (see Experimental Section). A 1:1 Langmuir model was used for curve fitting to a pseudo-first-order association kinetics and a single exponential dissociation kinetics (Fig. 4, see also Experimental section). Association of RyR2 peptide to CaM was relatively slow (*k*^on^ = 15 × 10^3^ M^−1^ s^−1^). The dissociation process was instead relatively fast (*k*^off^ = 7.1 × 10^–3^ s^−1^, which corresponds to a complex half-life time (*t*_1/2_) of 97.6 s). Interestingly, all LQTS-associated CaM variants showed significantly faster (1.5- to 12-fold) dissociation from the RyR2 peptide, the fastest dissociations being observed for the substitutions in His (*t*_1/2_ = 15.6 s for D95H and 8 s for D131H). The association process was generally less affected, except for D131E, which showed a fivefold faster association (Fig. 4). Two variants showed a completely different behavior. The association phase of D95H CaM did not follow any apparent trend, with a very fast increase followed by a fast drop of the signal. A possible explanation is the different conformation of this variant, as detected by NMR and CD spectroscopy, which could have interfered with the immobilization/orientation of the protein on the chip. On the other hand, no binding was detected for D129V CaM under the tested conditions, fully in line with ITC data, which showed that the binding mechanism is essentially different for this variant. Taken together, both ITC and SPR analyses suggest mutation-specific effects on CaM-RyR2 recognition. In particular, no general trend beside faster dissociation for all LQTS CaM variants could be obtained by SPR data. It is worth noting that caution should be used when interpreting ITC data. Indeed, the inferred thermodynamic parameters are assumed to refer to the pure Ca^2+^-protein binding process, but at least for Ca^2+^-sensor proteins, they have been shown to be significantly influenced by the thermodynamic contribution of protein conformational changes, which can be comparable or even exceed the energetic contributions of pure binding [35, 36]. The not optimal correlation between ITC and SPR data is, therefore, expected when monitoring this type of binding process.

### LQTS-associated CaM variants alter the topological properties of the protein-peptide structure network

Exhaustive and reproducible Molecular Dynamics (MD) simulations (Figs. S14, S15 and S16) allow the identification of persistent non-covalent interactions among amino acids, which define variant-specific protein structure networks (PSN) that can help explaining both local structural dynamics and long-range allosteric effects in Ca^2+^-sensor proteins [27, 30]. EF3 and EF4 CaM variants were, therefore, subjected to all-atom MD simulations to evaluate the rewiring of the network of persistent interactions and unveil potential allosteric effects associated with the amino acid substitutions. Interestingly, the analysis of the size of hydrophobic clusters of CaM-RyR2 complex highlighted a lower persistence of half-maximal hydrophobic clusters (pT) for D95 variants (22.1 for D95H and 18.8% for D95V vs. 24.2% for WT, Table S3) and D129V variant, which exhibited the largest decrease in pT (16.8%), highlighting a less favorable protein-peptide packing for this variant, in line with previous experimental evidence. On the contrary, mutations in D131 increased such persistence (26.7 for D131E and 26.2% for D131H, Table S3), suggesting that pT depends on the position of the mutation, rather than on the nature of the substitution, which may exert a tightening or loosening effect on the hydrophobic interaction network.

**Fig. 5:**
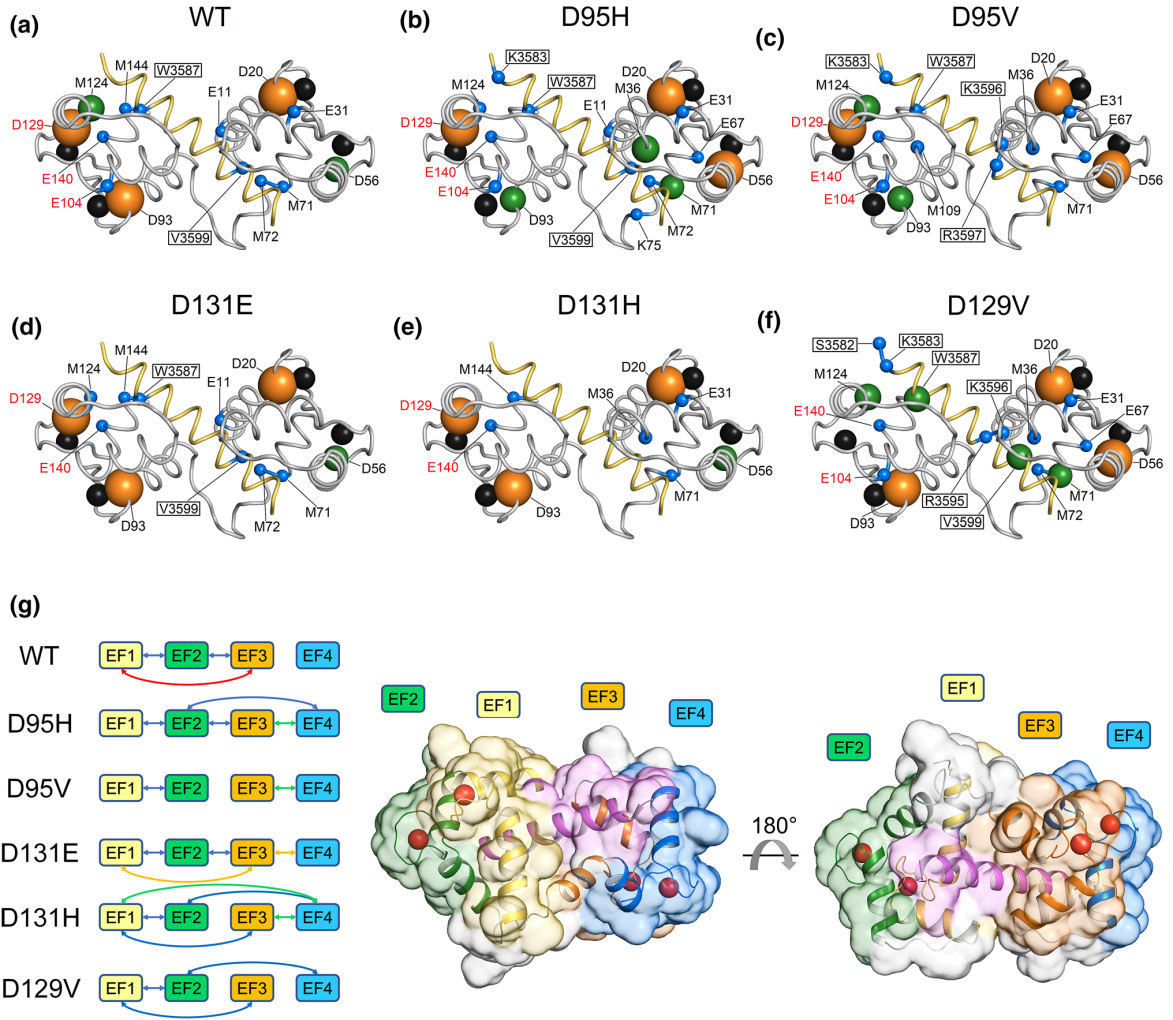
Protein-peptide Structure Network analysis based on Molecular Dynamics simulations. **a**–**f** High degree hubs of WT CaM-RyR2 complex and CaM variants D95H, D95V, D131E, D131H and D129V. Protein structure is shown as tubes, with CaM colored in grey and RyR2 peptide in yellow. Ca^2+^ ions are shown as black spheres. The Cα of hub residues with degree 6, 7 and 8 are represented as blue, green and orange spheres, respectively, with radius proportional to the degree of the hub. Residues with mutations associated with cardiac arrhythmias are labelled in red. Hub residues belonging to the RyR2 peptide are framed. **g** Diagram representing the most robust communication pathways connecting Glu residues, which constitute the bidentate Ca^2+^-coordinators of the 4 EF-hands in WT and mutated CaM-RyR2 complexes. EF1 is shown in yellow, EF2 in green, EF3 in blue and EF4 in orange. Blue arrows: communication robustness index (CR) > 0.1, green arrows: CR > 0.2, yellow arrows: CR > 0.3, red arrows: CR > 0.4 (individual values are reported in Fig. S16). Cartoon representation of the 3D structure of Ca^2+^-loaded CaM-RyR2 complex where proteins structure is represented as cartoon and, with EF1 colored in yellow, EF2 in green, EF3 in orange, EF4 in cyan and RyR2 peptide in pink. Ca^2+^ ions are shown as red spheres; the solvent-accessible surface of CaM-RyR2 complex is shown in transparency and colored according to each EF-hand

The topological analysis of the PSN of WT CaM-RyR2 complex highlighted that 8 out of 12 residues whose side-chain replacements are associated with arrhythmia [12] interacted with at least 4 other residues (Table S3) and could thus be defined as hubs (Degree = 4). Hubs are crucial centers for mediating intra- and intermolecular communication among amino acids and are in fact responsible for maintaining functional protein structure and dynamics. For such reason, a mutation of such hub residues would most likely exert a more detrimental effect on the entire network than a node with a lower degree (< 3 interactions). When residues with hub degree ≥ 6 were considered for PSN analysis of CaM-complexes, an interesting pattern was detected for LQTS-variants (Fig. 5a). D95H/V substitutions displayed a general and more consistent increase in hub degree both concerning CaM and RyR2 (Fig. 5b, c and S17). On the other hand, mutations in D131 overall decreased the connectivity of hub residues (Fig. 5d, e and S17) with a more marked effect of the D131H variant, which exhibited a larger decrease in connectivity in both CaM and RyR2 hubs. Finally, D129V CaM showed the largest variation in hub degree with respect to the WT, with a ∆Degree =  − 6, accompanied by a generalized increase in connectivity of all hub residues belonging to RyR2. Therefore, mutating residue D129, that constitutes a 8-degree (maximum degree) hub in all CaM variants may lead to a dramatic rearrangement of possible routes of intramolecular communication in CaM, and intermolecular communication with RyR2 peptide. This, together with the lower pT displayed by this variant, suggests that different types of interactions are involved in the stabilization of CaM-RyR2 complex, which indeed showed significantly different structural features in NMR experiments (Fig. 3c) and a substantial rearrangement of the hydrophobic core according to near UV CD spectroscopy (Fig. 1b). In a recent study [37], Wang et al. investigated the interaction of another LQTS-associated variant affecting the same position, namely D129G, with a peptide of the Ca_V_1.2 IQ domain. It was found that, besides completely losing the Ca^2+^ binding to EF4, the substitution led to the misfolding of the C-lobe, which no longer formed significant contacts with the IQ domain. Although the peculiarities observed with that mutant could be partly attributed to the substitution into glycine, which has higher conformational flexibility compared to other residues, and could partly depend on the specific interaction with the peptide, these findings are in line with the results from PSN analysis based on exhaustive MD simulations, and in particular the role of D129 as a major hub for intra- and inter-molecular communication.

Interestingly, most of the residues showing a ∆Degree ≠ 0 are localized in the N-terminal lobe of CaM and the spatially adjacent C-terminal portion of the peptide (residues 3595–3600). This points to an allosteric effect of the amino acid substitutions occurring in the CaM variants investigated in this study. The mutations are all located in the EF3 and EF4 motifs of the C-terminal lobe of CaM, therefore, structurally far from the N-terminal interface with the target, where the structural (anche senza structural) effects are observed.

### CaM variants alter communication between EF-hands and with the RyR2 target

Intramolecular communication among the four Ca^2+^-coordinating EF-hands was computed by considering all possible connections between the four Glu residues constituting the bidentate Ca^2+^- ligands in each EF-hand and calculating the Communication Robustness index (CR; see Experimental Section) which accounts for the number and length of the shortest paths connecting the EF-hands. The analysis highlighted a significantly robust intradomain communication between EF1 and EF2 (located in the N-lobe) in all CaM variants (Fig. 5g and S18). In addition, WT CaM exhibited interdomain communication between EF2 and EF3 and most significantly, between EF1 and EF3 (Fig. S18). Surprisingly, for CaM WT no robust communication pathway was found to connect any EF hand with EF4, at odds with all pathological variants, which presented either intradomain communication (from EF3 in D95V and D131E), interdomain communication (from EF2 in D129V) or both (from EF2 and EF3 in D95H, from all three EF-hands in D131H) (Fig. 5g). The isolation of EF4 from the other EF-hands motifs in the presence of the RyR2 target seems, therefore, to be characteristic of WT CaM, while its connection to close or allosterically far EF hands is a peculiarity of the LQTS-associated variants.

A characteristic of the EF3 variants is that D95H/V mutations completely abolish the robust communication between EF1 and EF3 shown by the WT (Fig. 5g) and still present in EF4 variants. Nevertheless, while D95H displayed communication between all neighboring EF-hands and an interdomain communication from EF2 to EF4, D95V exhibited only short-ranged communication among the EF-hands belonging to the same lobe, and the two domains lost the communication induced by the presence of the target. Concerning the mutations in EF4, D131H displayed the highest degree of interconnection among EF-hands, as EF4 was found to robustly communicate with all other EF-hands, while, at odds with the WT, no interdomain communication between adjacent EF2 and EF3 was detected. A WT-like behavior was instead shown by D131E variant in terms of both intra- and interdomain communication, with the addition of the short-ranged communication between EF3 and EF4, both located at the C-lobe. Finally, the D129V variant showed a different pattern of coupling between EF-hands: beside the EF1-EF2 intradomain communication in the N-lobe shared by all variants, a coupled interdomain communication between EF1 and EF3 and between EF2 and EF4 was detected in this case.

Not only the intramolecular communication among the Ca^2+^-binding motifs was found to be altered by the presence of mutations, but also the intermolecular communication with the target RyR2. Indeed, D95H variant showed an overall slightly decreased communication from EF1 to the CaM-RyR2 interface residues, while for D95V the average CR was almost halved with respect to the WT (Fig. S19). D131E substitution did not significantly impact the EF1-interface communication, whereas D131H displayed a larger reduction in CR compared to the WT, though with some exceptions (E47, E127, M144). D129V variant was found to be the most detrimental for the target-EF1 communication, as all interface residues exhibited a fourfold reduction in CR. This, together with the many peculiarities noticed for the D129V variants suggests a major role of the EF1-RyR2 communication for achieving the correct quaternary structure, and evidently to prepare the two molecules for the correct recognition.

At odds with the EF1-interface communication, all disease-associated variants enhanced the intermolecular communication with the target originating from EF2, though to a variant-specific extent (Fig. S19). The increase in CR in D95H and D129V variants involved interface residues belonging to different structural regions of the RyR2 target, whereas in D95V only the interface residues of the protein C-lobe exhibited significantly higher CR values with respect to the WT. D131E displayed only minor differences with the WT, while D131H increased the communication of EF2, especially with the N-terminal region of the interface, suggesting that the communication with EF2 depends on the properties of the substitution of residue D131, as previously shown for EF1-interface communication.

Communication between the C-lobe EF-hands and the RyR2 showed fewer surprising alterations. The overall EF3-interface communication was nearly halved by the two D95 substitutions (Fig. S19), which could be somehow expected as the mutations involve the third Ca^2+^-coordinating residue of EF3. D129V variant disrupted the EF3-interface communication, similarly to the effect exerted on EF1-interface communication, with CR values > twofold lower than the WT for all interface residues, while D131 variants did not display significant differences with the WT. Despite the short distance between the mutated side chains and the peptide N-terminal residues, the EF4-interface communication was the least affected by the D95 variants, with D95V being slightly more detrimental than D95H, although the differences in CR were generally very limited and involved the entire interface (Fig. S19). D129V, by affecting the first Ca^2+^-coordinating residue of EF4, weakened the communication with RyR2 interface, while D131 variants showed a mild increase in CR with respect to the WT with domain-specific effects, as D131E affected mainly the C-terminal region of the interface, while D131H displayed a larger increase in interface residues located in the N-lobe.

## Concluding remarks

A complex tripartite equilibrium between Ca^2+^, CaM and RyR2 target is required in cardiomyocytes to achieve a physiological regulation of the CICR process. This equilibrium relies on microscopic equilibria that may influence each other in a complex fashion [38]. Slight perturbations of such equilibria can alter the homeostasis in cardiomyocytes and result in disease phenotypes at a macroscopic level. In this study, we used a combination of approaches to add new evidence on the impact of some LQTS- associated missense mutations in the perturbation of functional recognition between CaM and RyR2. We have shown that LQTS-associated mutations in EF3 and EF4 affect both CaM secondary and tertiary structure to a different extent. Despite the structural perturbations of each variant, it is at the level of protein-target interaction that common alterations emerge, suggesting some general characteristics of the arrhythmia-associated CaM variants analyzed in this study.

Interestingly, our data suggest that, despite slight alterations, the overall conformation of the CaM-RyR2 complex is maintained in the presence of the LQTS-associated missense mutations, yet all mutants showed faster dissociation from the target peptide compared to WT CaM. We, therefore, hypothesize that the variants could interfere with the regulation of the CICR process by altering the dynamics underlying the target recognition. We here demonstrate that, while showing a relatively low affinity for Ca^2+^ in the isolated protein [2], the N-terminal lobe is involved in allosteric interactions which appear to be essential for functional CaM-RyR2 recognition in the presence of Ca^2+^. This is demonstrated by the severe perturbation of intermolecular communication between EF2 and the interface with RyR2, observed for all LQTS mutants analyzed in this study, independent of the position of the mutation, and by the severely decreased EF1-RyR2 communication shown by the D129V CaM variant, for which the binding process was especially unfavorable and hardly detectable. EF1-RyR2 communication might, therefore, be a prerequisite not only for achieving the correct quaternary structure, but also to prepare the two proteins for correct recognition. These findings are in line with a recently proposed mechanism of conformational selection in CaM-target recognition, according to which long-range electrostatic interactions between the target and charged residues at the N-terminal initiate the binding while short-range, mostly hydrophobic interactions between the target and C-lobe residues in CaM determine selectivity [39].

The general consistency of our structural, thermodynamic, and kinetic characterization and results from all-atom MD simulations suggest that a similar analysis should be extended to other arrhythmia-associated CaM and/or RyR2 variants to infer general features of pathogenic CaM-RyR2 complexes and distinguish them from functional ones.

## Supplementary Information

Below is the link to the electronic supplementary material.

Supplementary file1 (PDF 17145 KB)

## Data Availability

Data and material are available upon request.
