# Supplementary material for: Alterations in calmodulin-cardiac ryanodine receptor molecular recognition in congenital arrhythmias
Source: Cell Mol Life Sci. 2022 Feb 8;79(2):127. doi: 10.1007/s00018-022-04165-w (PMC8825638; doi:10.1007/s00018-022-04165-w)
Supplement: Supplementary file 1 — Supplementary file1 (PDF 17145 KB) [file 18_2022_4165_MOESM1_ESM.pdf]

SUPPLEMENTARY INFORMATION

Alterations in calmodulin-cardiac ryanodine receptor molecular recognition in congenital arrhythmias

Giuditta Dal Cortivo, Carlo Giorgio Barracchia, Valerio Marino, Mariapina D'Onofrio and Daniele Dell'Orco

Supplementary Figures

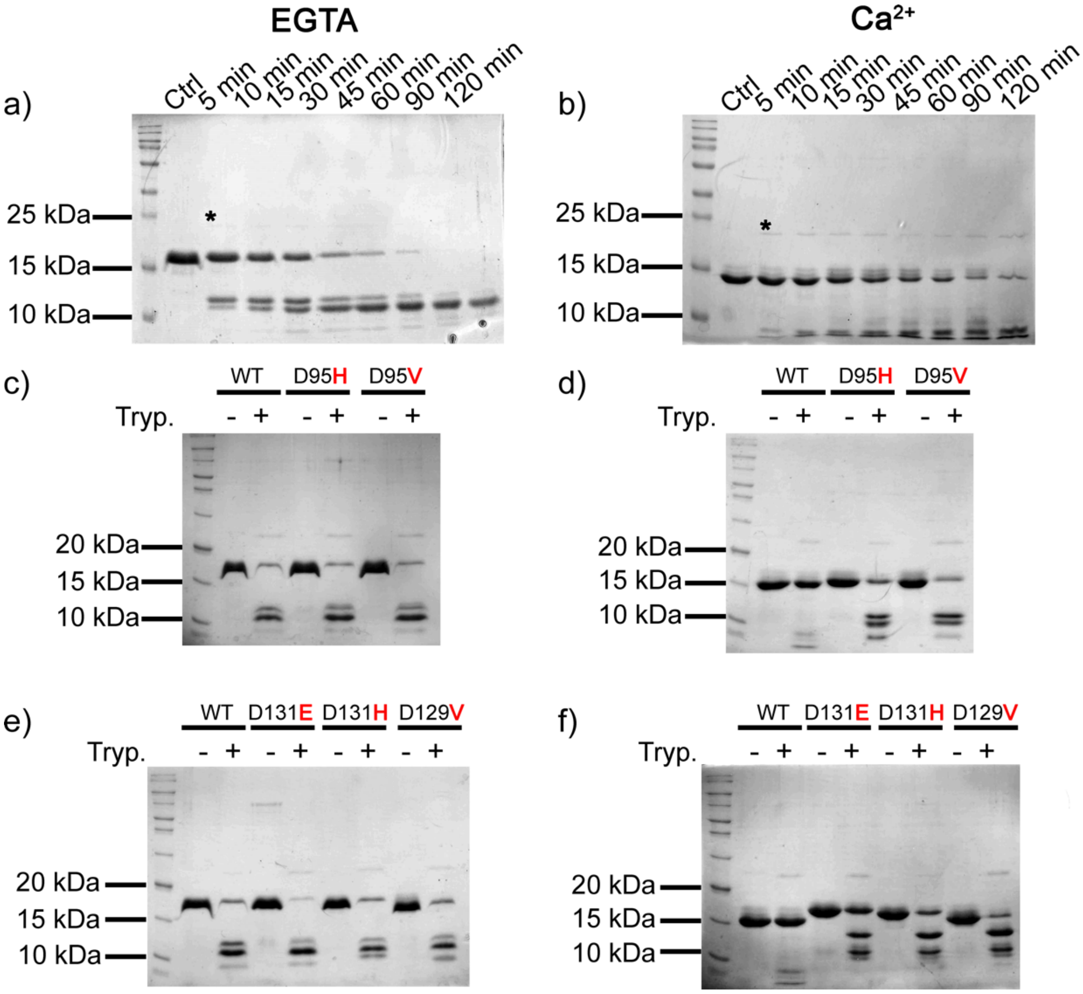

Figure S1. Limited proteolysis profiles. a-b) Time scan proteolysis following incubation of trypsin with 20 μM CaM WT (ratio 1:60) in the presence of 2 mM EGTA (a) or Ca^{2+} (b) at 25°C. The band marked with an asterisk corresponds to trypsin. c-f) Limited proteolysis profiles after 10 minutes incubation of CaM variants in the presence of EGTA (c, e) or Ca^{2+} (d, f). Undigested samples were included as reference. Samples were boiled for 10 minutes and Coomassie Blue stained.

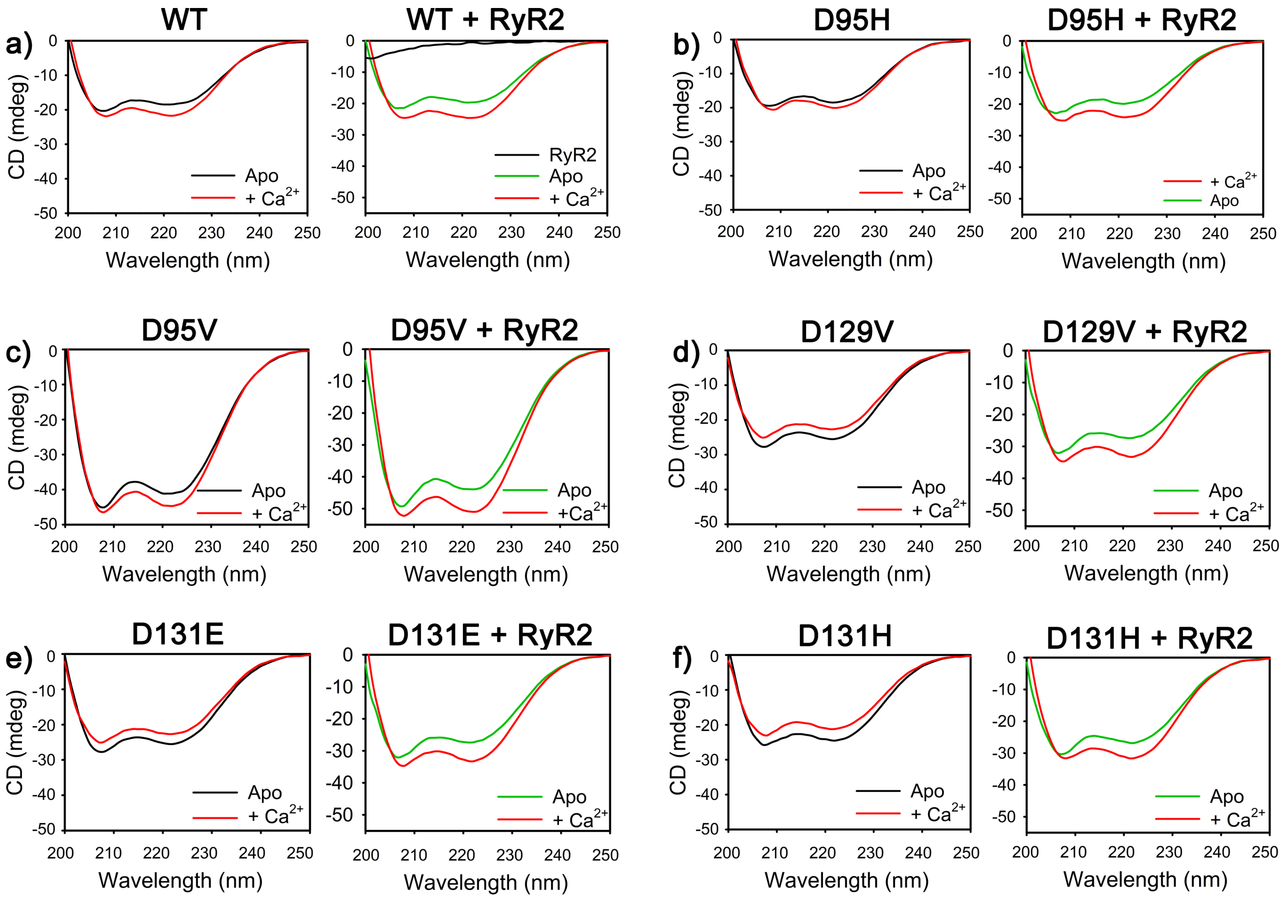

Figure S2. Secondary structure of CaM variants investigated by far UV CD spectroscopy. Far UV spectra of isolated CaM variants (left columns) were collected in the presence of 300 μM EGTA (black lines) and after the addition of 600 μM Ca^{2+} (left columns, red lines). To investigate the structural contribution of RyR2 (right columns), spectra of obtained after mixing 10 μM CaM and 20 μM RyR2 were collected without Ca^{2+} (apo, green lines) and after the addition of 600 μM Ca^{2+} (right columns, red lines). The spectrum of the sole RyR2 peptide shown in WT + RyR2 panel (black) was recorded to show the lack of secondary structure in the absence of its target. Spectra were collected at 25°C in the 200-250 nm range (time response = 4 s), using a 0.1 cm pathlength quartz cuvette. The spectrum of the buffer was recorded and considered as a reference, thus subtracted from protein spectra. Each spectrum represents the average of 5 accumulations.

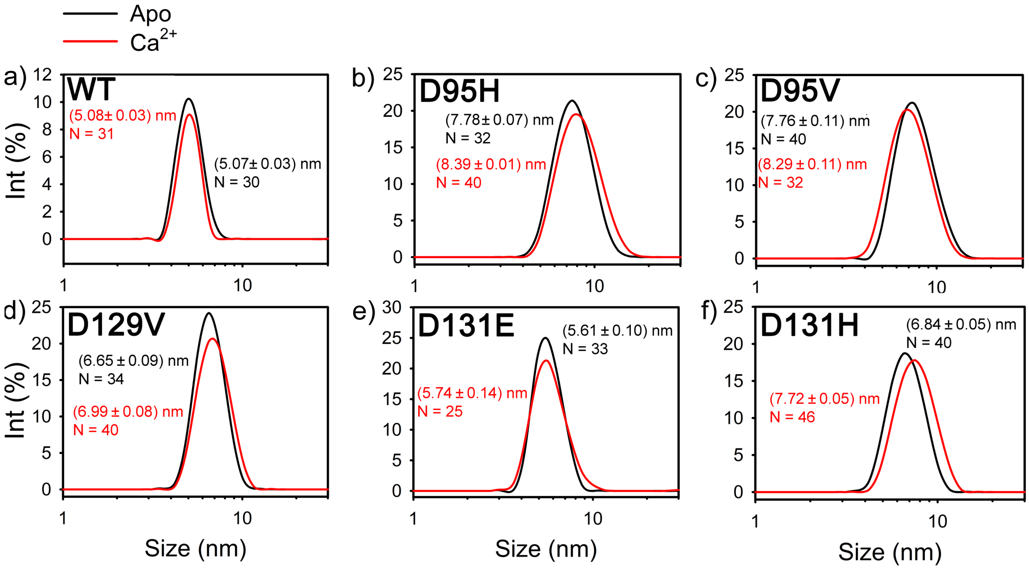

Figure S3. Hydrodynamic size of CaM variants investigated by Dynamic Light Scattering. Dynamic light scattering of 30 mM of each CaM variant in the presence of 1 mM EGTA (black lines) or 2 mM Ca^{2+} (red lines) after filtration through a 20 nm membrane. Each panel shows the number of measurements (N) and the hydrodynamic diameter as mean ± s.e.m (standard error of the mean).

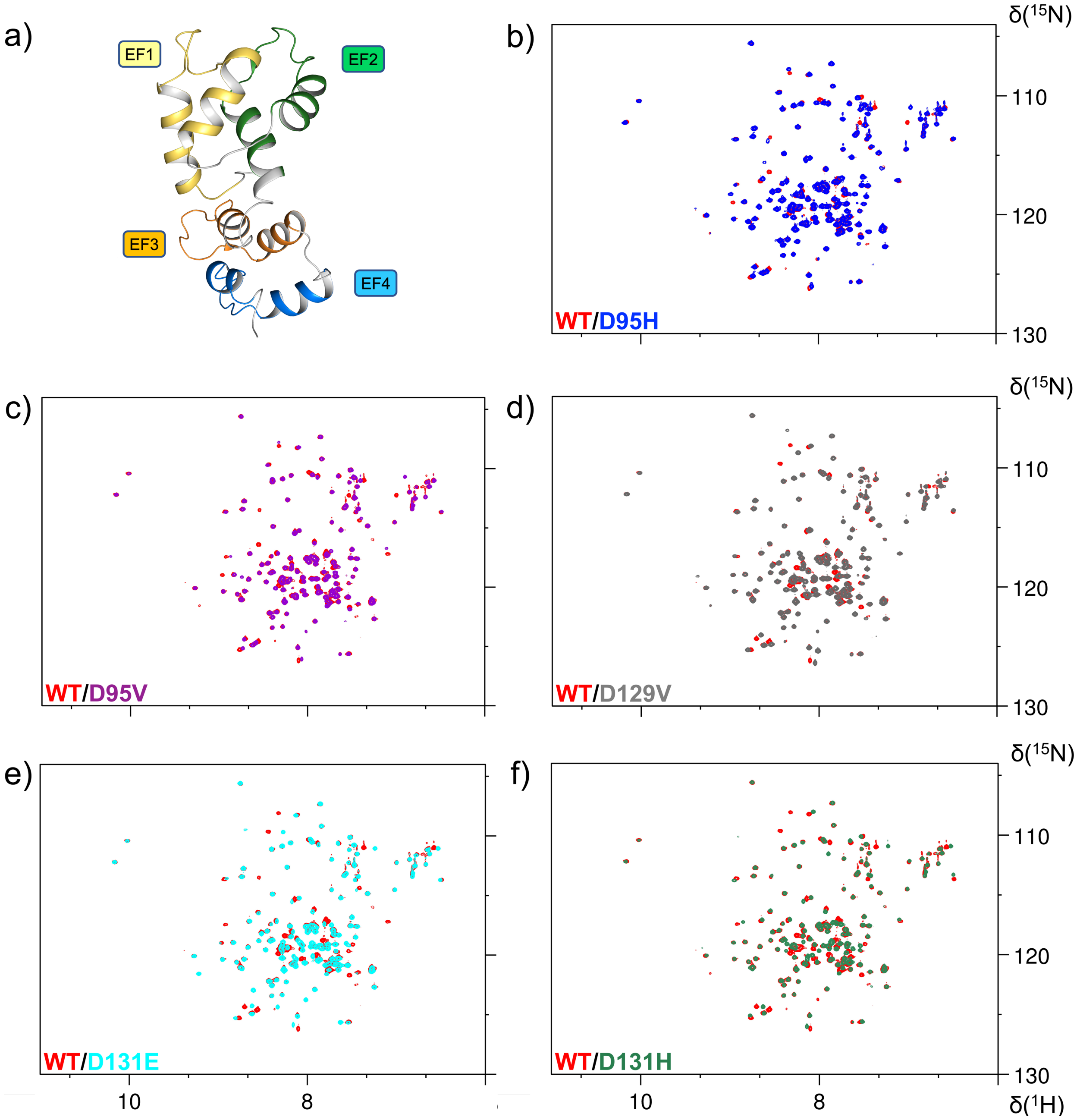

Figure S4. Bidimensional NMR spectra of CaM variants. a) Three-dimensional structure of apo-CaM (PDB entry: 1DMO). b-f) Overlay of the ^{1}H-^{15}N HSQC NMR spectra of s4tyhjk^{15}N-WT CaM (red) and its variants in their Ca-free forms.

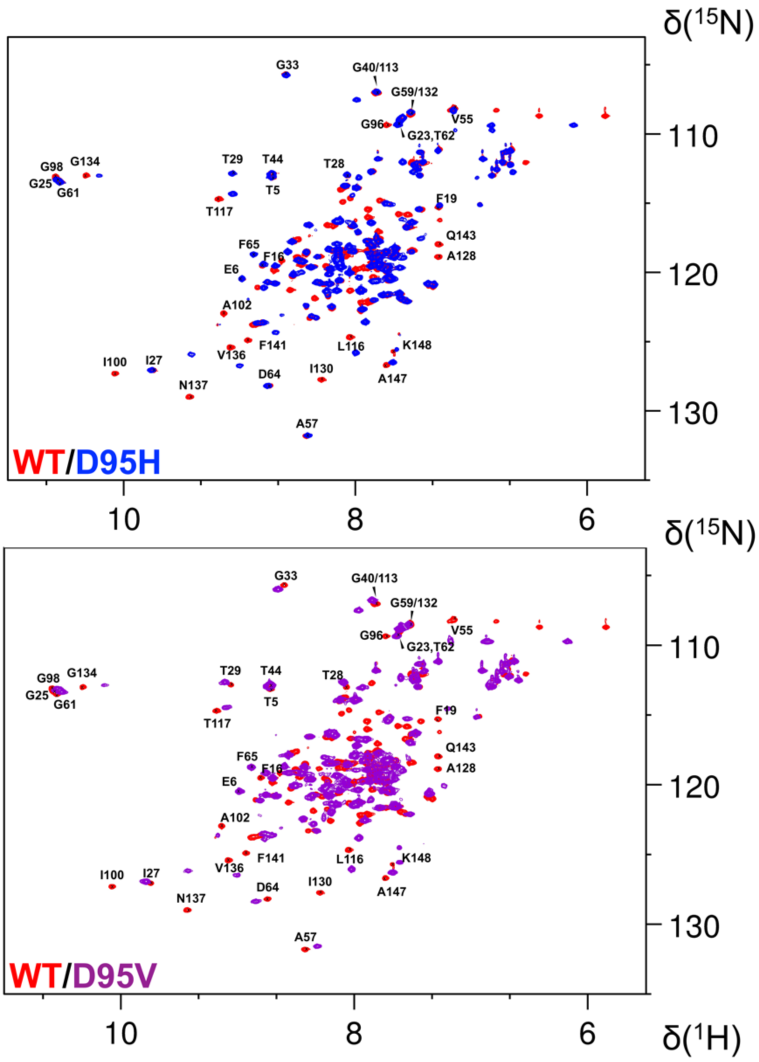

Figure S5. Bidimensional NMR spectra of CaM variants. a-b) Overlay of the ^{1}H-^{15}N HSQC NMR spectra of ^{15}N-WT CaM (red) and D95 variants in the presence of 2 mM Ca^{2+} ions. The labels indicate the assignment of the residues in the spectrum (red) of the Ca^{2+} bound WT CaM.

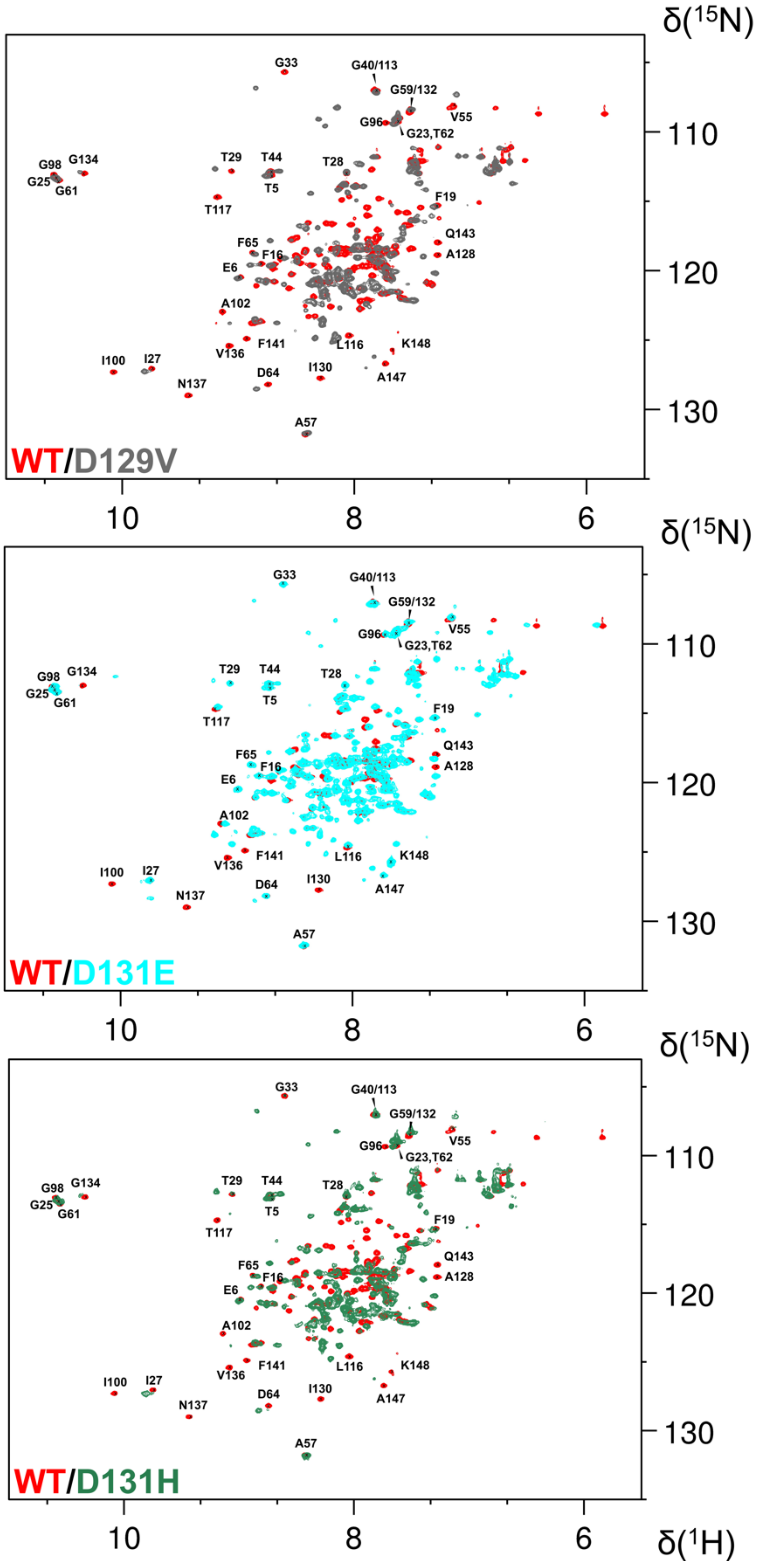

Figure S6. Bidimensional NMR spectra of CaM variants. a-c) Overlay of the ^{1}H-^{15}N HSQC NMR spectra of ^{15}N-WT CaM (red) and D129V (a) or D131 (b and c) variants in the presence of 2 mM Ca^{2+} ions. The labels indicate the assignment of the residues in the spectrum (red) of the Ca^{2+} bound WT CaM.

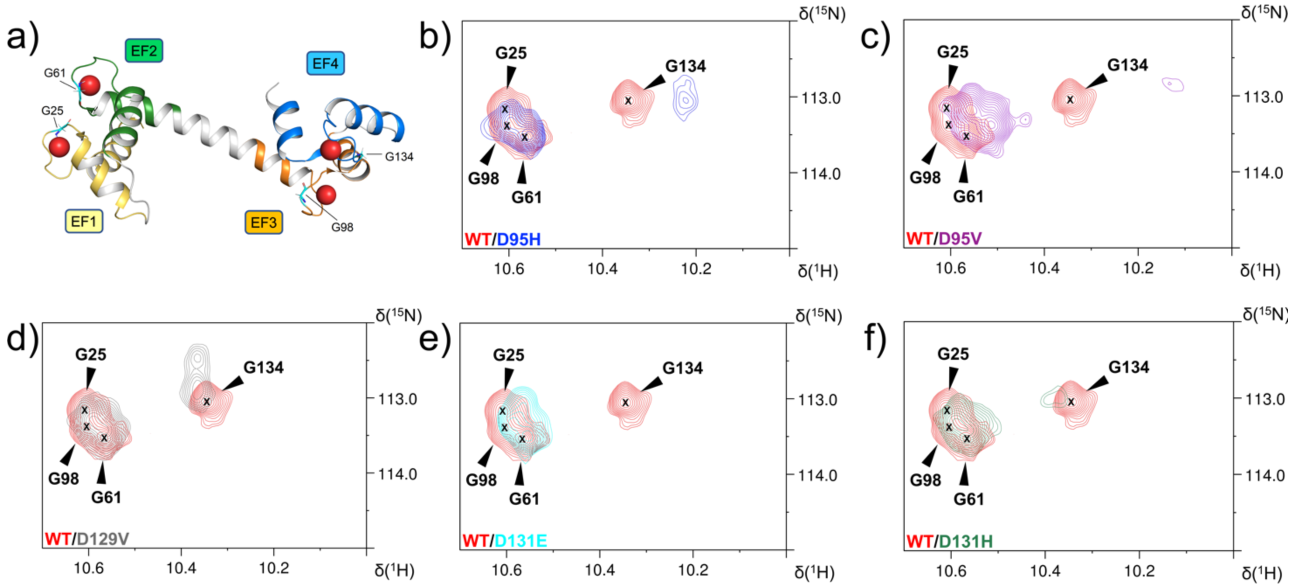

Figure S7. Comparison of a selected region of ^1H-^{15}N HSQC NMR spectra showing peaks of the residues G25, G134, G98, and G61. a) Three-dimensional structure of Ca^{2+}-loaded CaM (PDB entry: 1CLL[1]). Protein structure is represented as cartoons, with EF1 colored in yellow, EF2 in green, EF3 in orange and EF4 in blue. Ca^{2+} ions are shown as red spheres, G25, G61, G98 and G134 shown in the NMR spectra are depicted as cyan sticks. b-f) Overlay of the downfield region of the ^1H-^{15}N HSQC spectra of ^{15}N-WT CaM and its variants in the presence of 2 mM Ca^{2+} ions.

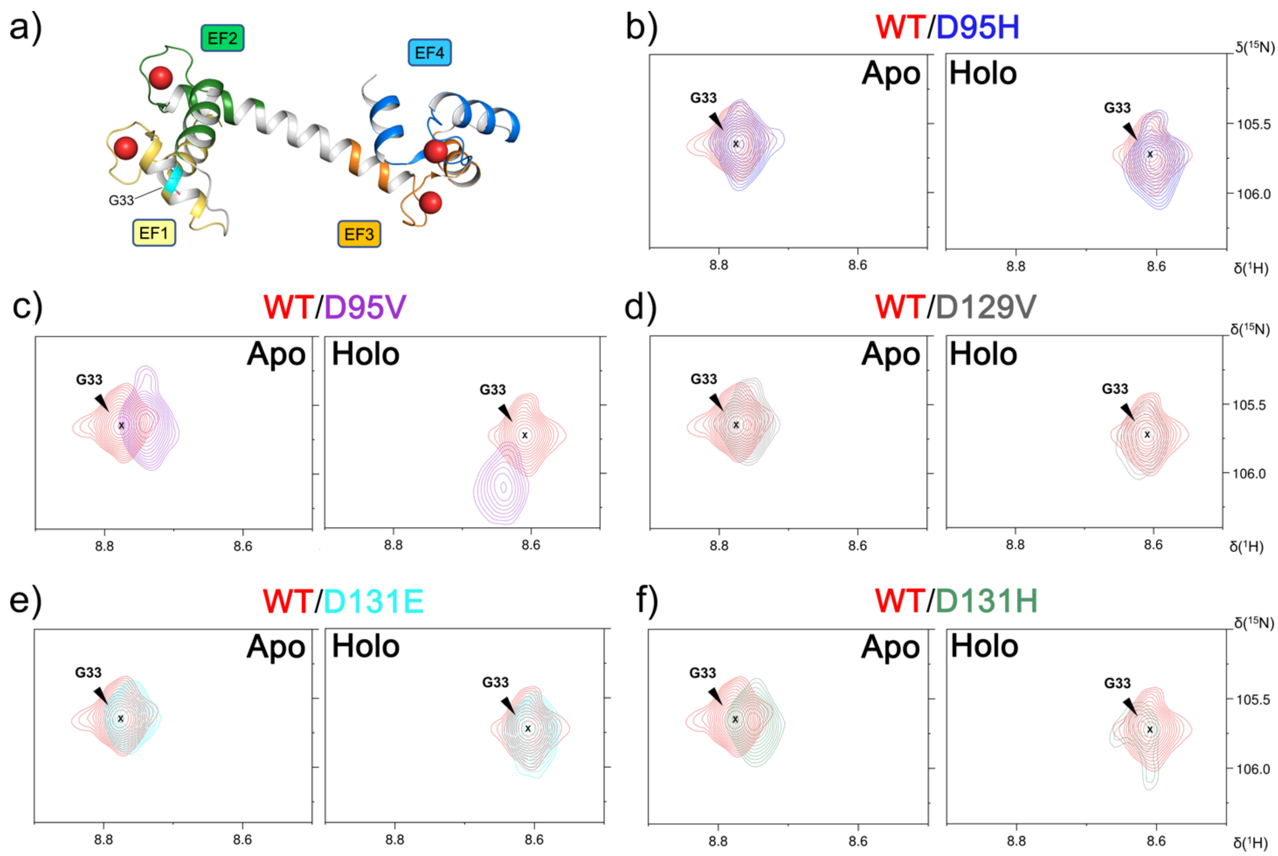

Figure S8. Comparison of a selected region of ^1H-^{15}N HSQC spectra showing peaks of the residue G33. a) Three-dimensional structure of Ca^{2+}-loaded CaM (PDB entry: 1CLL). Protein structure is represented as cartoons, with EF1 colored in yellow, EF2 in green, EF3 in orange and EF4 in blue. Ca^{2+} ions are shown as red spheres, G33 shown in the NMR spectra is depicted as cyan sticks. b-f) Close-up view of the overlaid ^1H-^{15}N HSQC spectra of ^{15}N-WT CaM and its variants, in their Apo and Ca^{2+}-bound (Holo) forms.

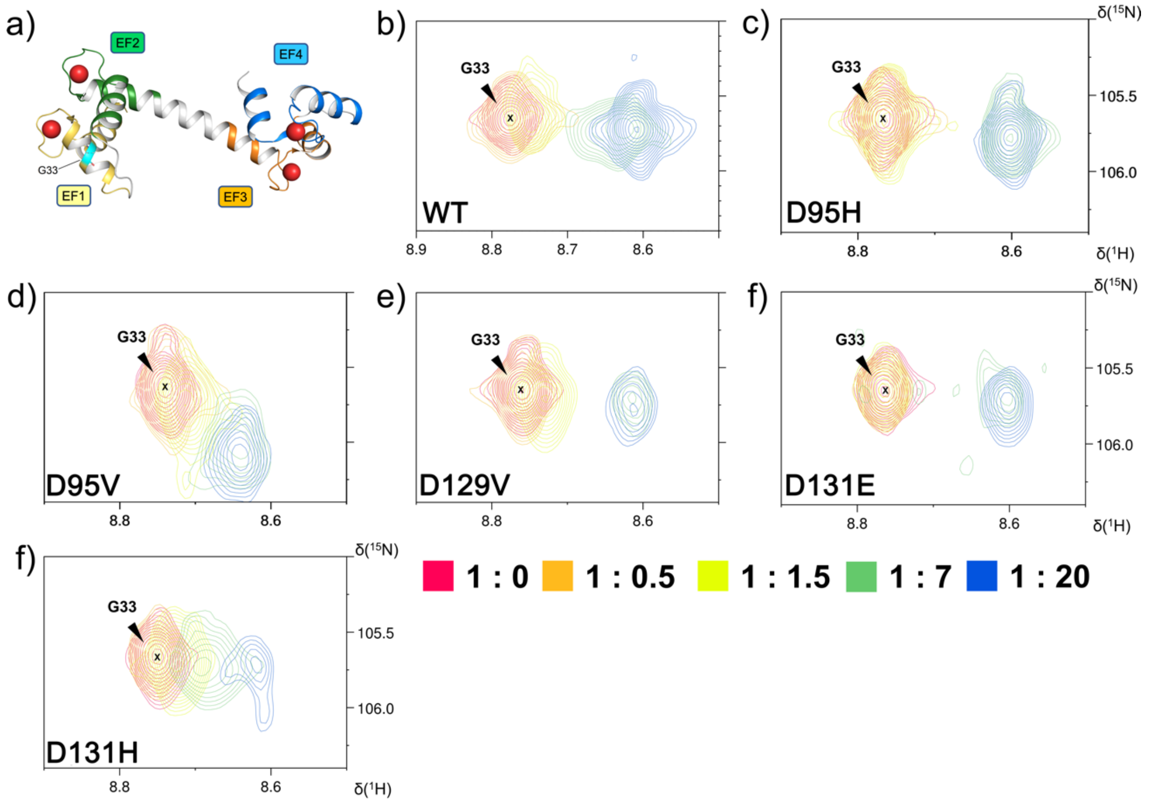

Figure S9. Differential behavior of NMR signals of G33 of ^{15}N-CaM variants upon addition of Ca^{2+} ions. a) Three-dimensional structure of Ca^{2+}-loaded CaM (PDB entry: 1CLL). Protein structure is represented as cartoons, with EF1 colored in yellow, EF2 in green, EF3 in orange and EF4 in blue. Ca^{2+} ions are shown as red spheres, G33 shown in the NMR spectra is depicted as cyan sticks. b-f) Close-up views of the ^{1}H-^{15}N SOFAST HMQC spectra of 300 µM ^{15}N-CaM and variants showing signal shifts of G33 as a function of addition of Ca^{2+} ions.

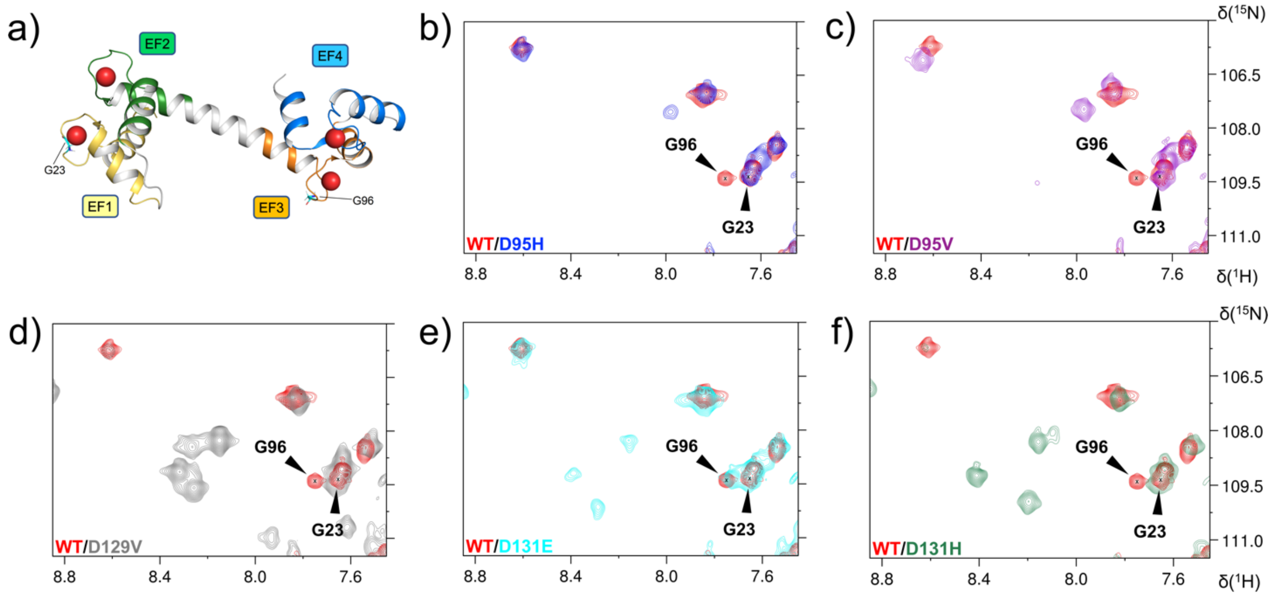

Figure S10. Differential behavior of NMR signals of G23 and G96 of ^{15}N-CaM variants upon addition of Ca^{2+} ions. a) a) Three-dimensional structure of Ca^{2+}-loaded CaM (PDB entry: 1CLL). Protein structure is represented as cartoons, with EF1 colored in yellow, EF2 in green, EF3 in orange and EF4 in blue. Ca^{2+} ions are shown as red spheres, G23 and G96 shown in the NMR spectra are depicted as cyan sticks. b-f) Close-up views of the ^{1}H-^{15}N SOFAST HMQC spectra of ^{15}N-CaM and variants showing signal shifts of G23 and G96 as a function of addition of Ca^{2+} ions. Protein concentration was 300 µM.

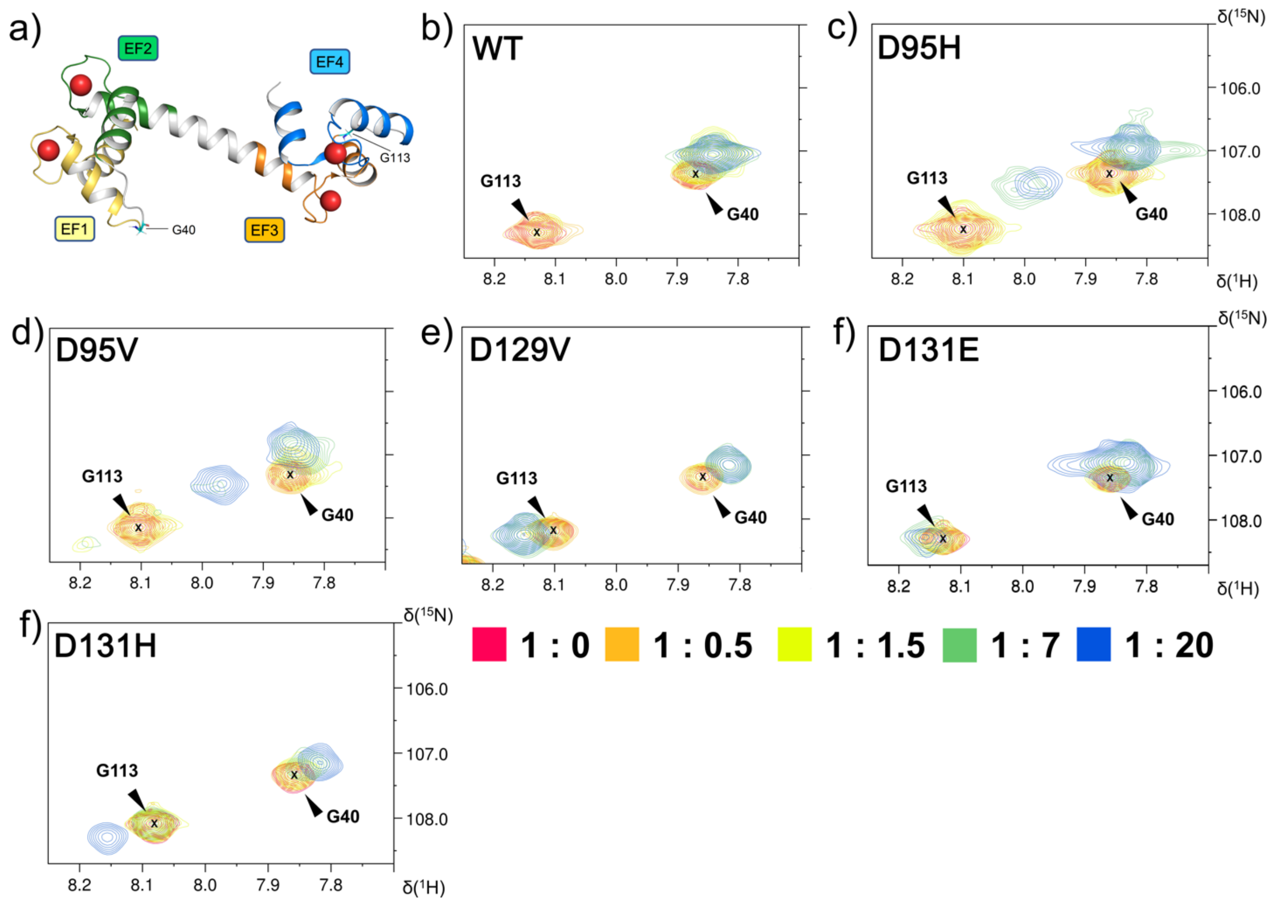

Figure S11. Differential behavior of NMR signals of G40 and G113 of ^{15}N-CaM variants upon addition of Ca^{2+} ions. a) Three-dimensional structure of Ca^{2+}-loaded CaM (PDB entry: 1CLL). Protein structure is represented as cartoons, with EF1 colored in yellow, EF2 in green, EF3 in orange and EF4 in blue. Ca^{2+} ions are shown as red spheres, G40 and G113 shown in the NMR spectra are depicted as cyan sticks. b-f) Close-up views of the ^{1}H-^{15}N SOFAST HMQC spectra of 300 μM ^{15}N-CaM and variants showing signal shifts of G40 and G113 as a function of addition of Ca^{2+} ions.

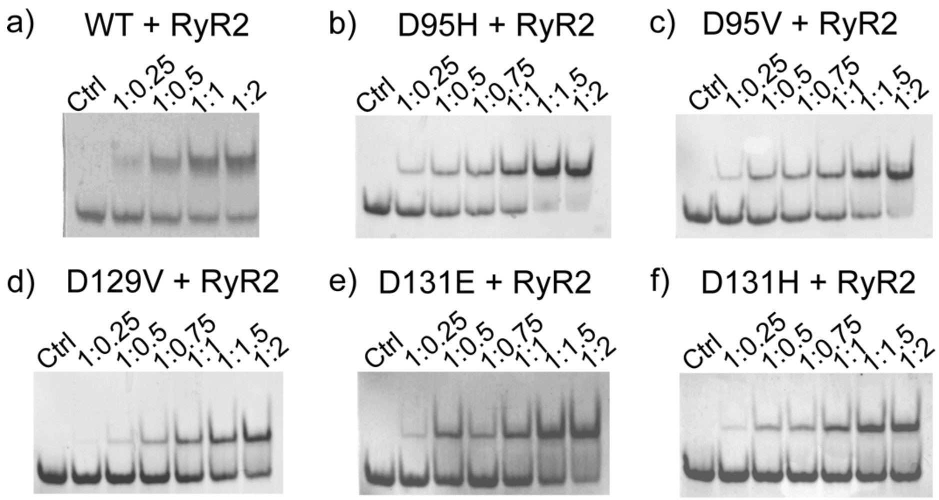

Figure S12: Stoichiometry of CaM:RyR2 interaction assessed by non-denaturing PAGE. CaM variants (17 μM) were incubated with increasing concentrations of RyR2 (1:0 to 1:2 CaM:RyR2 ratios) for 10 minutes at 25°C using 20 mM TRIS pH 7.5, 150 mM KCl, 1 mM DTT, 340 μM Ca^{2+} as working buffer. Samples were loaded on continuous 15% polyacrylamide gels, run at 200 V for 45 min and Coomassie Blue stained.

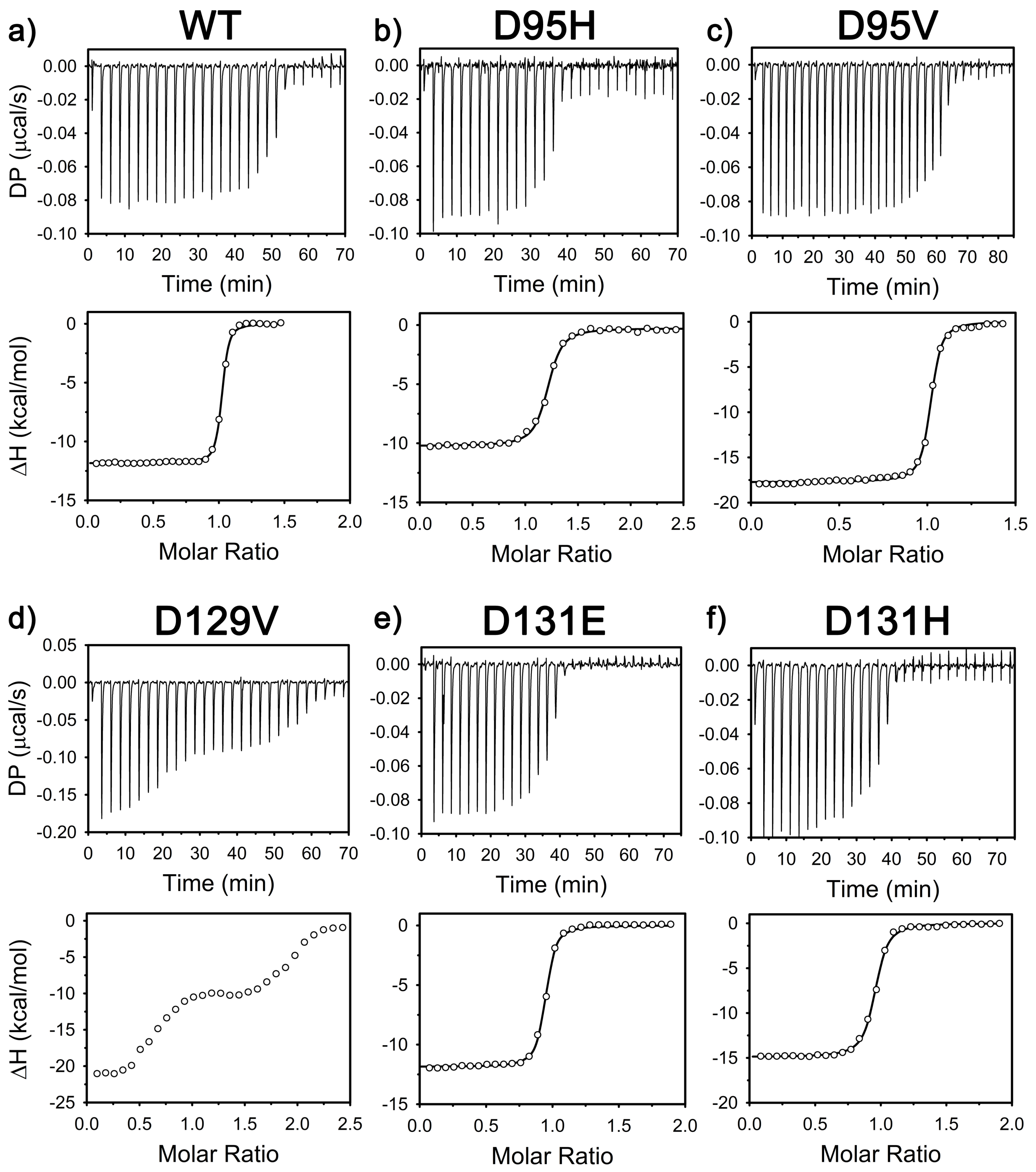

Figure S13. Thermodynamic properties of CaM-RyR2 interaction monitored by Isothermal Titration Calorimetry. ITC measurements were performed by titrating RyR2 (125 μM, in the titrant syringe) on 7.5 μM (panels b) and d)) or 10 μM CaM variants (cell). Thirty 1 μL-injections were performed setting the stirring velocity to 750 rpm and allowing a 150 s interval between injections. Twenty millimolar TRIS pH 7.5, 150 mM KCl, 5 mM Ca^{2+} was used as working buffer. Thermodynamic parameters obtained by 3-6 replicas are shown in Table S2. Panels report an example of titration curves obtained for each variant.

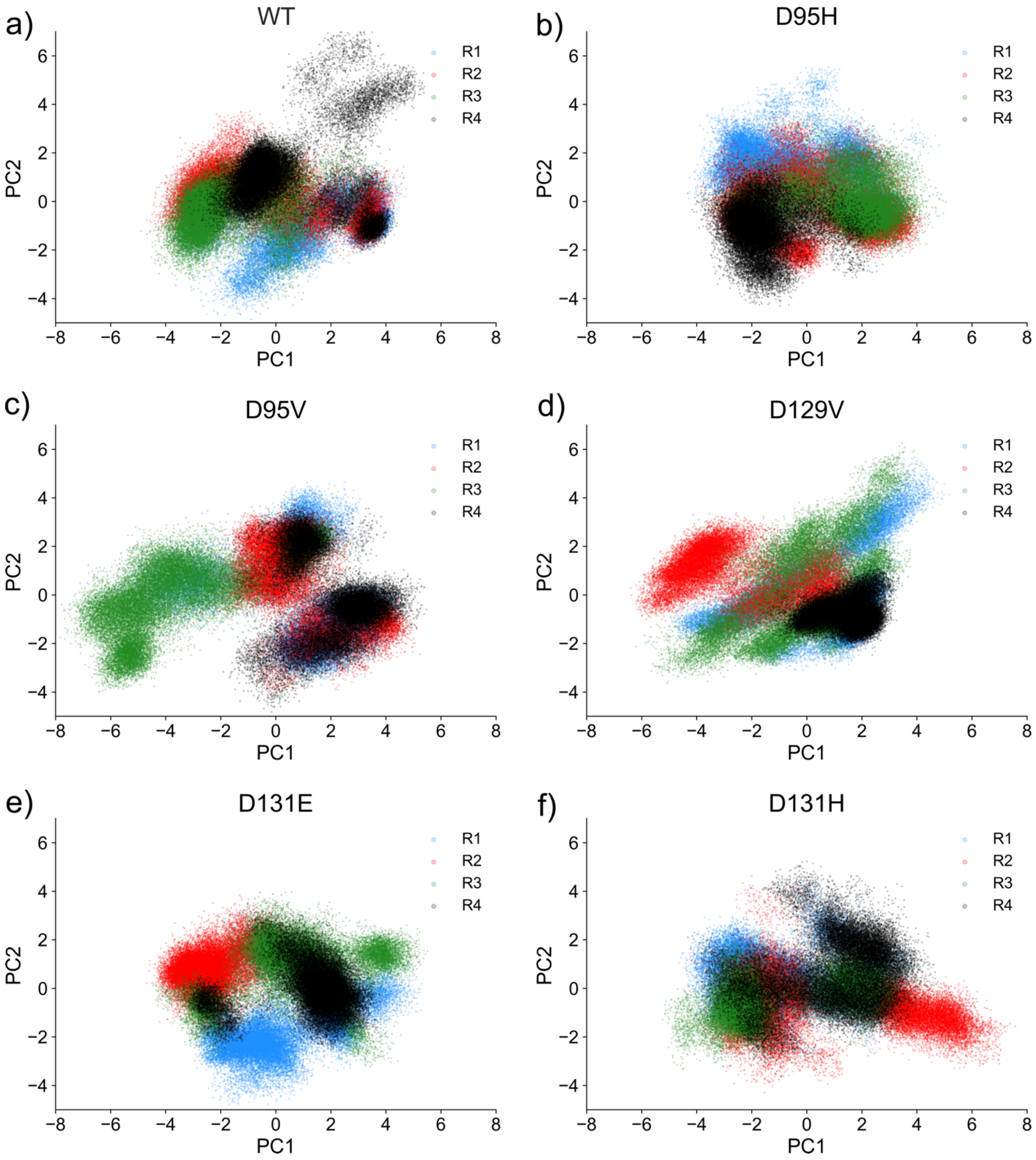

Figure S14. **Reproducibility test of MD simulations independent replicas - 1**. Projections of the frames of the four 300 ns MD simulation replicas (R1 to R4) onto the first two Principal Components (PC1 and PC2) calculated on the concatenated 1.2 μs trajectories of WT CaM-RyR2 complex and CaM variants D95H, D95V, D129V, D131E and D131H. Replica R1 frames are colored in blue, R2 in red, R3 in green and R4 in black.

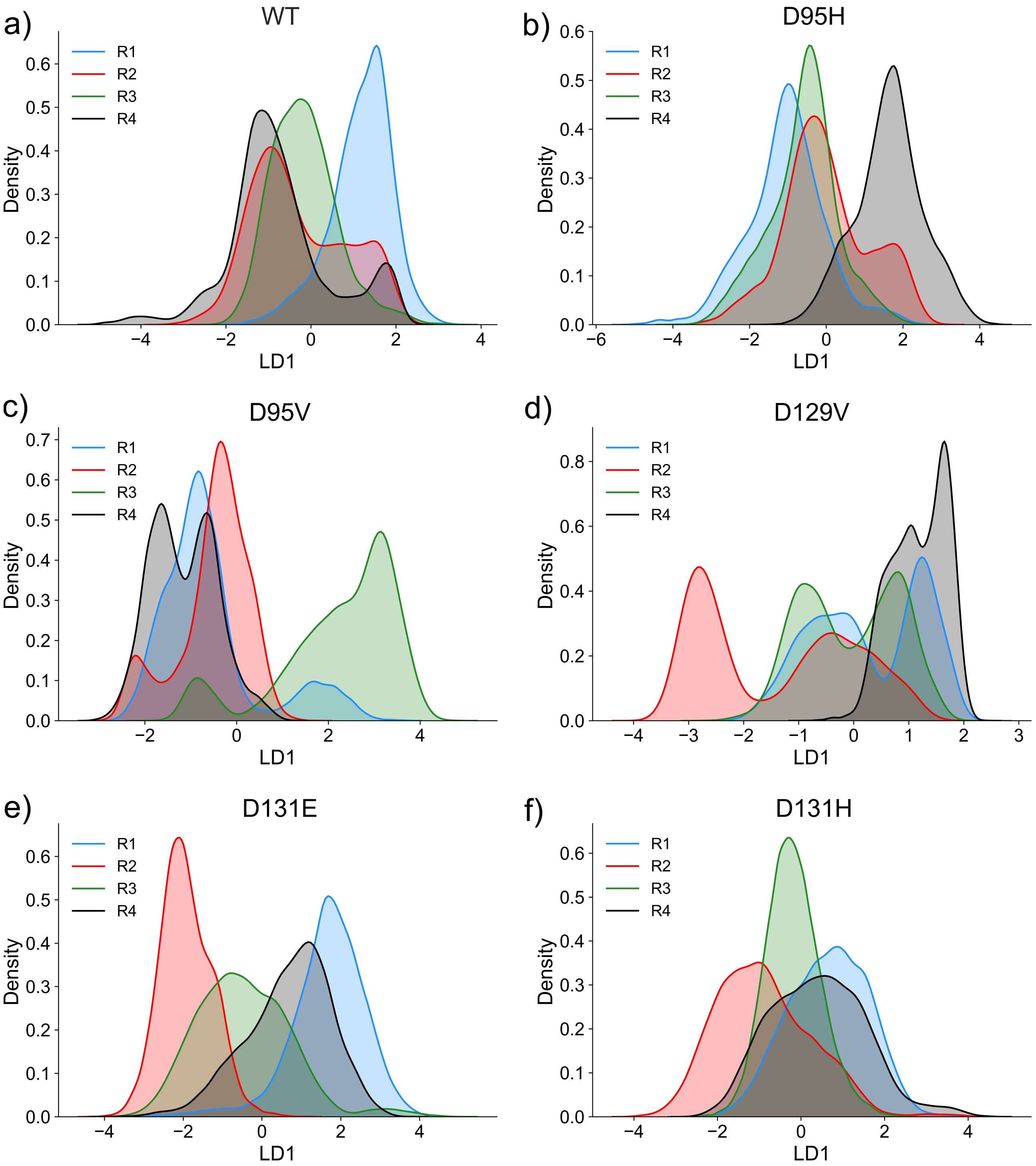

Figure S15. **Reproducibility test of MD simulations independent replicas – 2.** Linear Discriminant Analysis of the projections of the frames of the four 300 ns MD simulation replicas (R1 to R4) onto the first two Principal Components calculated from the concatenated 1.2 μs trajectories of WT CaM-RyR2 complex and CaM variants D95H, D95V, D129V, D131E and D131H. Density of replica R1 frames projected onto LD1 are colored in blue, R2 in red, R3 in green and R4 in black.

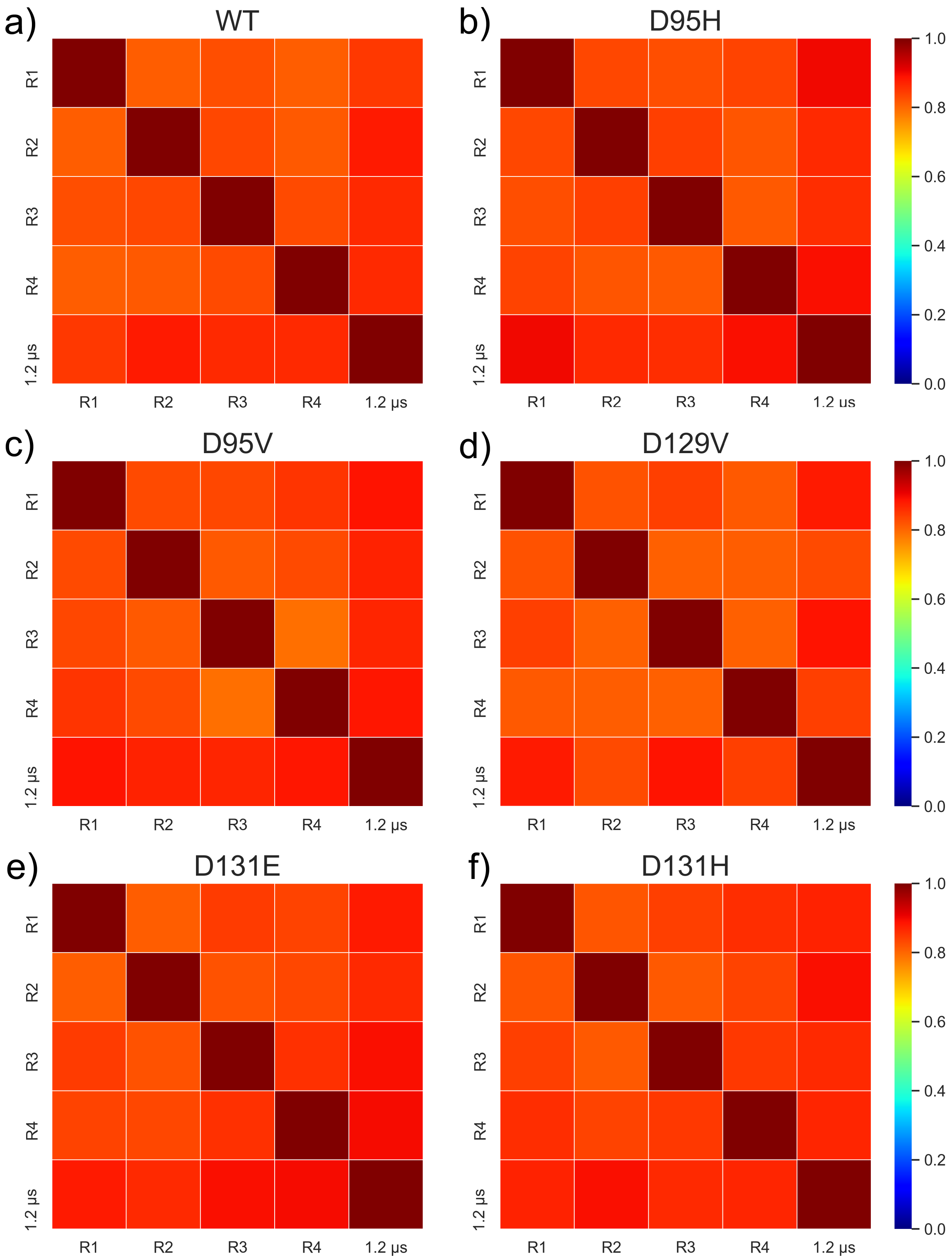

Figure S16. **Reproducibility test of MD simulations independent replicas – 3.** Root-Mean Square Inner Product (RMSIP) of the first 20 PC extracted from each of the four 300 ns and the concatenated 1.2 μs trajectories of the six CaM variant-RyR2 complex. RMSIP values were calculated in an all-vs-all fashion and represented in a blue-to-red (0 to 1) color scale.

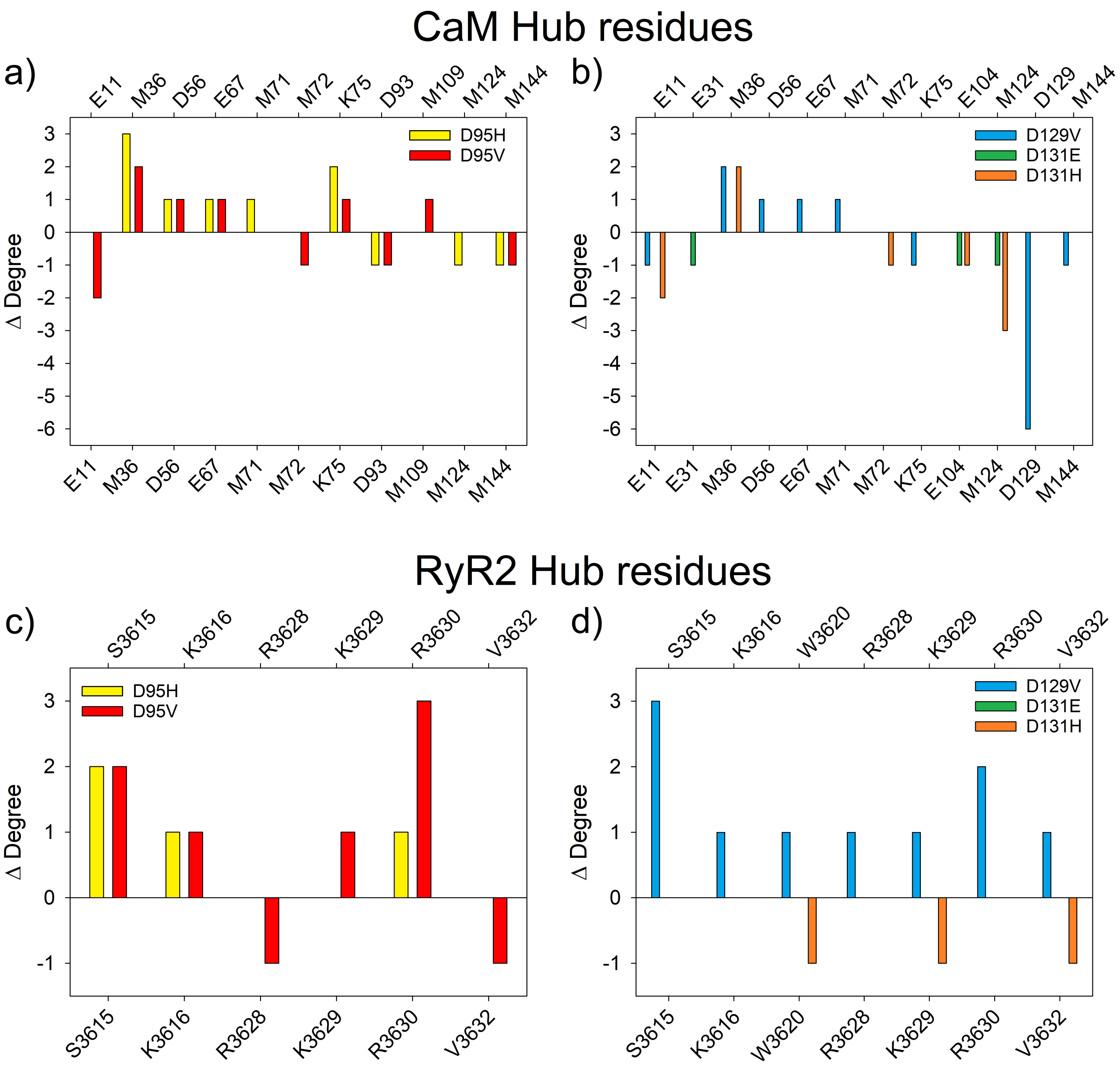

Figure S17. Effects of cardiac arrhythmia-associated variants on the connectivity of CaM (top panels) and RyR2 (bottom panels) hub residues in CaM-RyR2 complex. Residues were considered hubs if their degree was ≥ 6 in at least 1 variant (Table ST3). ΔDegree is the difference between the degree of the hubs in the variant (D95H yellow, D95V red, D129V blue, D131E green, D131H orange) and in the WT complex.

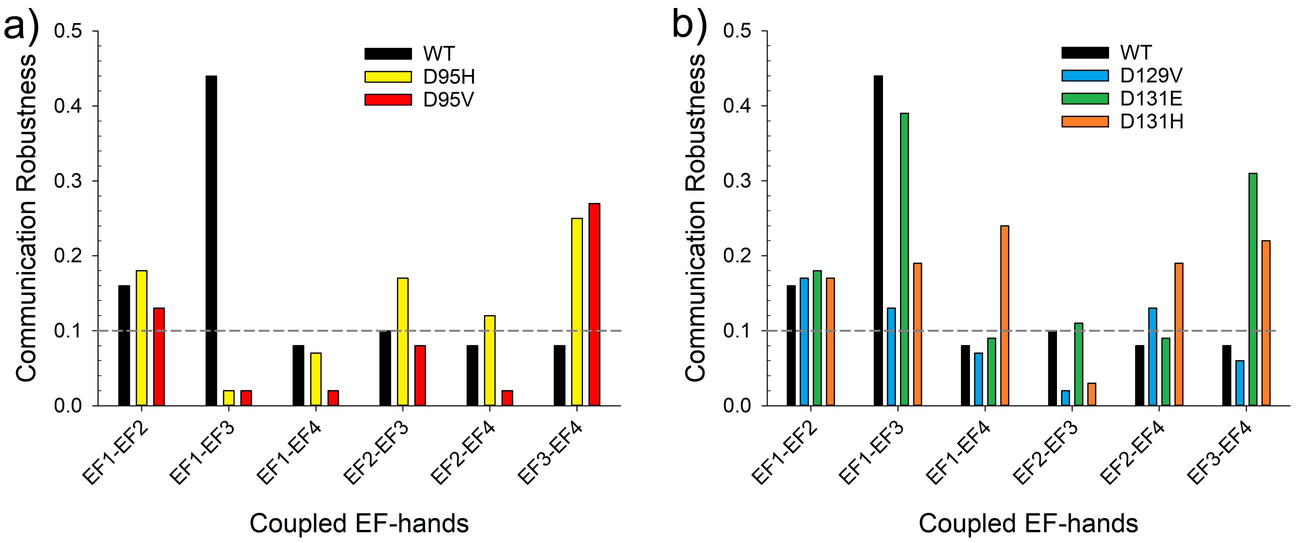

Figure S18. Robustness of intramolecular communication between EF-hands in CaM variants. Communication Robustness among representative Glu residues of EF1 to EF4 in WT CaM-RyR2 complex (black) and CaM variants D95H (yellow), D95V (red), D129V (blue), D131E (green), D131H (orange).

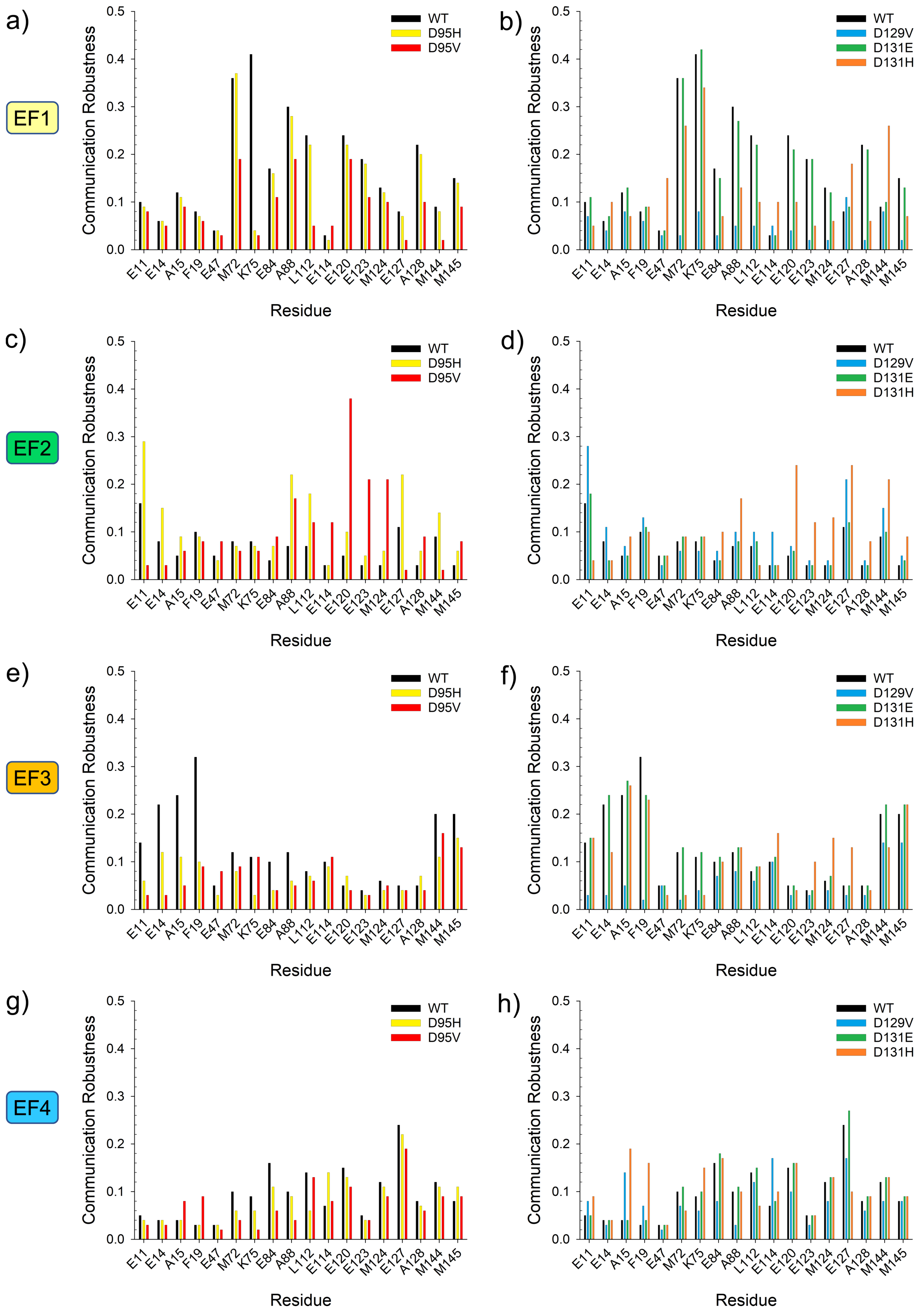

Figure S19. Robustness of intermolecular communication between EF-hands in CaM variants and RyR2 interface. Communication Robustness between representative Glu residues of EF1 to EF4 and CaM residues belonging to the RyR2 binding interface in WT CaM-RyR2 complex (black) and CaM variants D95H (yellow), D95V (red), D129V (blue), D131E (green), D131H (orange).

Supplementary Tables

Table S1. Thermodynamic parameters of CaM-RyR2 interaction measured by ITC. See the main text for a description of each quantity.

Table S2: Thermodynamic parameters measured via ITC for the binding of CaM variant with RyR2						
	N (# sites)	K_D (nM)	ΔH (kcal/mol)	ΔG (kcal/mol)	-TΔS (kcal/mol)	ΔΔG (kcal/mol)
**WT N = 5**	0.96 ± 0.03	8.62 ± 2.20	-11.28 ± 0.62	-11.01	0.27	-
**D95H N = 5**	1.06 ± 0.05	11.33 ± 5.62	-12.06 ± 1.03	-10.85	1.21	0.16
**D95V N = 4**	1.03 ± 0.01	4.78 ± 0.86	-15.35 ± 0.89	-11.36	3.99	-0.35
**D129V N = 9**	n.d.	n.d.	n.d.	n.d.	n.d.	n.d.
**D131E N = 3**	0.87 ± 0.03	14.17 ± 3.03	-11.37 ± 0.90	-10.70	0.67	0.31
**D131H N = 3**	0.89 ± 0.02	34.00 ± 2.40	-15.03 ± 0.03	-10.20	4.84	0.81

Table S2. Structural characterization of CaM variants by far UV CD spectroscopy. See the main text for a description of each quantity.

Table S1: CaM structural features investigated by CD			
CaM		$\theta_{222}/\theta_{208}$	Δθ/θ (%)
**WT**	Apo<br>+ Ca^{2+}	0.93<br>0.97	14
	RyR2 – Apo<br>+ Ca^{2+}	0.90<br>0.98	25
**D95H**	Apo<br>+ Ca^{2+}	0.95<br>0.98	9
	RyR2 – Apo<br>+ Ca^{2+}	0.88<br>0.96	22
**D95V**	Apo<br>+ Ca^{2+}	0.91<br>0.96	8.8
	RyR2 – Apo<br>+ Ca^{2+}	0.89<br>0.98	16
**D129V**	Apo<br>+ Ca^{2+}	0.92<br>0.91	-11.3
	RyR2 – Apo<br>+ Ca^{2+}	0.87<br>0.96	21.7
**D131E**	Apo<br>+ Ca^{2+}	0.92<br>0.91	-11
	RyR2 – Apo<br>+ Ca^{2+}	0.87<br>0.96	13.4
**D131H**	Apo<br>+ Ca^{2+}	0.96<br>0.92	-13.6
	RyR2 – Apo<br>+ Ca^{2+}	0.89<br>1.00	17.8

Table S3. Protein Structure Network analysis resulting from MD simulations. Persistence of hydrophobic clusters (pT) was calculated as in Ref. [2], CaM residues associated with cardiac arrhythmias are shown in bold, RyR2 residues are underlined, Ca^{2+} ions are reported with the identifier of the EF-hand they are bound to and shown in italic.

**Table S3: Hubs of the PSN of WT CaM-RyR2 complex and CaM variants D95H, D95V, D129V, D131E and D131H**												
**Variant (pT %)**	**WT (24.2)**		**D95H (22.1)**		**D95V (18.8)**		**D129V (16.8)**		**D131E (26.7)**		**D131H (26.2)**	
Degree	Hubs		Hubs		Hubs		Hubs		Hubs		Hubs	
8	D20	D93	D20	D56	D20	D56	D20	D56	D20	D93	D20	D93
	**D129**		**D129**		**D129**		D93		**D129**		**D129**	
7	D56	M124	M36	M71	D93	M124	M71	M124	D56		D56	
			D93				W3587	V3599				
6	E11	E31	E11	E31	E31	M36	E31	M36	E11	M71	E31	M36
	M71	M72	E67	M72	E67	M71	E67	M72	M72	M124	M71	**E140**
	**E104**	**E140**	K75	**E104**	**E104**	M109	**E104**	**E140**	**E140**	M144	M144	
	M144	W3587	M124	**E140**	**E140**	K3583	S3582	K3583	W3587	V3599		
	V3599		K3583	W3587	W3587	K3596	R3595	K3596				
			V3599		R3597							
5	F16	D64	F16	D64	F16	V35	E11	F16	F16	E31	F16	E67
	E67	A73	A73	A88	M72	K75	V35	V55	E67	**E104**	M72	**E104**
	**F89**	M109	M109	V121	**F89**	V121	D64	**F89**	M109	I125	M109	I125
	V121	I125	I125	E127	I125	E127	M109	V121	E127	A128	A128	K3583
	E127	A128	A128	M144	A128	M144	I125	E127	K3583	R3595	W3587	R3595
	N137	K3583	S3582	R3595	S3582	K3593	A128	**D131**	K3596		V3599	
	R3595	K3596	K3596		V3599	V3600	M144	V3586				
					R3604		K3593	R3597				
							V3600	F3603				
							R3604					
4	E14	A15	E14	A15	E11	E14	E7	Q8	A15	D22	Q8	E11
	F19	D22	D22	D24	A15	L18	I9	E14	D24	T26	E14	A15
	D24	T26	T26	V35	F19	D22	A15	F19	V35	M36	D22	D24
	T34	V35	V55	D58	D24	T26	D22	D24	V55	D58	T26	V35
	M36	V55	T62	F68	L32	Q41	T26	L32	T62	D64	V55	D58
	D58	T62	T70	R74	E47	Q49	E47	L48	F68	A73	T62	D64
	F68	K75	E87	**F89**	D50	I52	Q49	D50	K75	A88	F68	A73
	A88	R90	R90	F92	E54	V55	D58	T62	**F89**	R90	K75	E87
	**D95**	L105	R106	**D131**	D58	T62	F68	T70	**D95**	L105	A88	**F89**
	R106	**D131**	**D133**	N137	D64	F68	A73	E87	R106	**F141**	R90	**D95**
	**D133**	**F141**	Y138	**F141**	T70	A73	A88	R90	M145	V3586	V121	M124
	M145	V3586	K3584	V3586	E87	A88	**D95**	S101	L3591	R3604	E127	**F141**
	L3591	R3604	L3591	R3597	R90	F92	L105	R106	*Ca1*	*Ca4*	M145	V3586
	Ca1	*Ca2*	V3600	F3603	L105	R106	E123	**D133**			L3591	K3593
	Ca4		R3604	*Ca1*	**D131**	**D133**	**F141**	K3584			K3596	V3600
			Ca2	*Ca4*	N137	**F141**	K3589	L3591			R3604	*Ca1*
					K3584	V3586	A3598	*Ca1*				
					L3591	R3595	*Ca2*					
					A3598	A3601						
					Ca1	*Ca4*						

Supplementary References

[1] Chattopadhyaya, R., Meador, W. E., Means, A. R., and Quiocho, F. A. (1992) Calmodulin structure refined at 1.7 A resolution, *J Mol Biol 228*, 1177-1192.

[2] Marino, V., and Dell'Orco, D. (2016) Allosteric communication pathways routed by Ca(2+)/Mg(2+) exchange in GCAP1 selectively switch target regulation modes, *Sci Rep 6*, 34277.

[3] Gong, D., Chi, X., Wei, J., Zhou, G., Huang, G., Zhang, L., Wang, R., Lei, J., Chen, S. R. W., and Yan, N. (2019) Modulation of cardiac ryanodine receptor 2 by calmodulin, *Nature 572*, 347-351.
